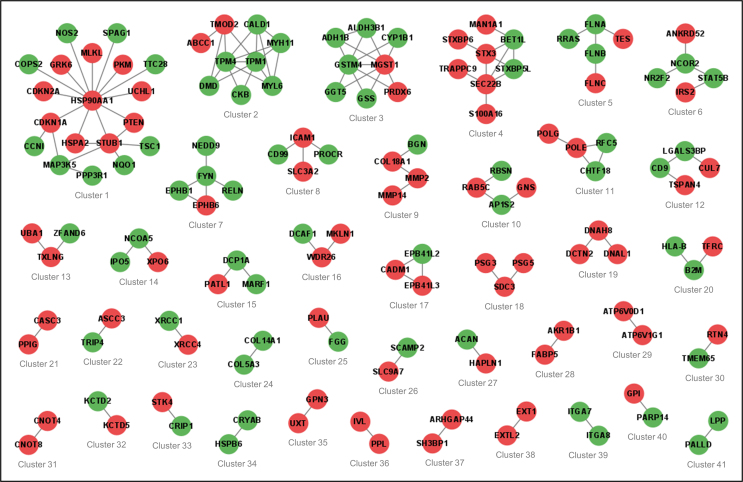# Quantitative proteome analysis of LAP1-deficient human fibroblasts: A pilot approach for predicting the signaling pathways deregulated in LAP1-associated diseases

**DOI:** 10.1016/j.bbrep.2024.101757

**Published:** 2024-06-26

**Authors:** Cátia D. Pereira, Guadalupe Espadas, Filipa Martins, Anne T. Bertrand, Laurent Servais, Eduard Sabidó, Philippe Chevalier, Odete A.B. da Cruz e Silva, Sandra Rebelo

**Affiliations:** a Institute of Biomedicine (iBiMED), Department of Medical Sciences, University of Aveiro, 3810-193, Aveiro, Portugal; b Center for Genomics Regulation, The Barcelona Institute of Science and Technology (BIST), Barcelona, Spain; c Universitat Pompeu Fabra, Barcelona, Spain; d Sorbonne Université, Inserm, Institut de Myologie, Centre de Recherche en Myologie, Paris, France; e MDUK Oxford Neuromuscular Center, Department of Paediatrics, University of Oxford and NIHR Oxford Biomedical Research Center, Oxford, OX3 9DU, United Kingdom; f Neuromuscular Center, Division of Paediatrics, University Hospital of Liège and University of Liège, 4000, Liège, Belgium; g Université Claude Bernard Lyon 1, Lyon, France; h Hospices Civils de Lyon, Lyon, France

**Keywords:** Proteomics, Mass spectrometry, Bioinformatics, DNA repair, Protein synthesis, Proteostasis, Oxidative stress response

## Abstract

Lamina-associated polypeptide 1 (LAP1), a ubiquitously expressed nuclear envelope protein, appears to be essential for the maintenance of cell homeostasis. Although rare, mutations in the human LAP1-encoding *TOR1AIP1* gene cause severe diseases and can culminate in the premature death of affected individuals. Despite there is increasing evidence of the pathogenicity of *TOR1AIP1* mutations, the current knowledge on LAP1's physiological roles in humans is limited; hence, investigation is required to elucidate the critical functions of this protein, which can be achieved by uncovering the molecular consequences of LAP1 depletion, a topic that remains largely unexplored. In this work, the proteome of patient-derived LAP1-deficient fibroblasts carrying a pathological *TOR1AIP1* mutation (LAP1 E482A) was quantitatively analyzed to identify global changes in protein abundance levels relatively to control fibroblasts. An *in silico* functional enrichment analysis of the mass spectrometry-identified differentially expressed proteins was also performed, along with additional *in vitro* functional assays, to unveil the biological processes that are potentially dysfunctional in LAP1 E482A fibroblasts. Collectively, our findings suggest that LAP1 deficiency may induce significant alterations in various cellular activities, including DNA repair, messenger RNA degradation/translation, proteostasis and glutathione metabolism/antioxidant response. This study sheds light on possible new functions of human LAP1 and could set the basis for subsequent in-depth mechanistic investigations. Moreover, by identifying deregulated signaling pathways in LAP1-deficient cells, our work may offer valuable molecular targets for future disease-modifying therapies for *TOR1AIP1*-associated nuclear envelopathies.

## Highlights

• Proteome analysis of LAP1-deficient patient's cells reveals dysfunctional processes.
• DNA repair, translation, proteostasis and oxidative stress response are deregulated.
• LAP1 is required for the maintenance of genome integrity in a physiological state.
• Potential therapeutic targets for LAP1-associated diseases are uncovered.

## Graphical abstract

**Figure:**
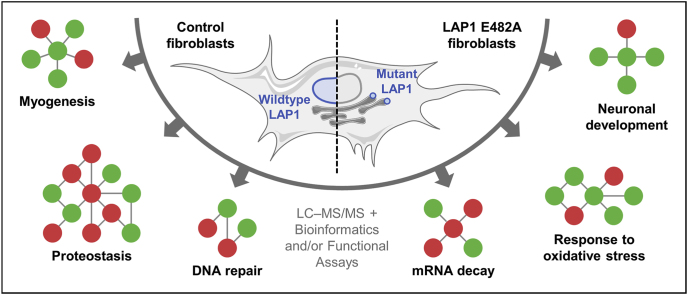

## 1 Introduction

Lamina-associated polypeptide 1 (LAP1) is a ubiquitous type II integral membrane protein that resides in the nuclear envelope (NE) [[1], [2], [3]]. Two human LAP1 isoforms are encoded by the *TOR1AIP1* gene, namely LAP1B [3] and LAP1C, which exhibit a tissue- and developmental stage-specific expression [4], suggesting distinct functional properties. To date, LAP1 has been proposed to participate in diverse biological processes, such as somatic cell division [5,6], DNA damage response (DDR) [7], cell migration [8], spermiogenesis [9], myogenic differentiation [10], skeletal muscle maintenance and growth [11,12], neuromuscular transmission [13], regulation of heart's left ventricular function [14] and hepatic very-low-density lipoprotein (VLDL) secretion [15]. These findings inspire an emerging view of LAP1 as a highly versatile protein that plays a role in many cellular activities and one can anticipate a long way to fully understand its physiological relevance.

In the last decade, several recessively inherited pathogenic *TOR1AIP1* mutations leading to complete or partial loss of LAP1 protein expression have been identified in 23 individuals around the world. These rare genetic alterations have been causatively linked to extremely incapacitating and often life-threatening clinical conditions, varying from tissue-selective disorders that affect primarily the skeletal muscle, heart and/or brain (e.g. muscular dystrophy, myasthenic syndrome, cardiomyopathy and dystonia) [13,[16], [17], [18], [19], [20], [21], [22]] to a complex multisystemic pathology that extends to numerous tissues [23,24]. Of note, this association of loss-of-function *TOR1AIP1* mutations with the development of nuclear envelopathies [13,[16], [17], [18], [19], [20], [21], [22], [23], [24]], allied to the fact that *Tor1aip1*^*−/−*^ mice display perinatal lethality [25], is another indicator of the paramount importance of LAP1 for normal cellular functioning. Moreover, it has recently been found that *TOR1AIP1* expression is significantly altered in various tumor types [26] and that LAP1 upregulation can contribute to an enhanced migratory and invasive ability of melanoma cells [27], which indicates that LAP1 may also be involved in cancer. Altogether, there is considerable evidence pointing to the necessity of deciphering human LAP1 (dys)function.

Of particular interest for this work, in 2014, Dorboz and colleagues reported the case of a Moroccan male child diagnosed with severe dystonia, cerebellar atrophy and dilated cardiomyopathy, who carried a homozygous missense *TOR1AIP1* mutation (c.1448A>C) that resulted in the substitution of a highly conserved amino acid in the C-terminal domain of both human LAP1 isoforms (p.E482A). The *in vitro* characterization of patient-derived skin fibroblasts revealed a substantial decrease in the protein levels of LAP1B and LAP1C in comparison to control skin fibroblasts, accompanied by a marked reduction or full absence of mutant LAP1 in the NE as well as its mislocalization and aggregation in the endoplasmic reticulum (ER) [17]. Regarding possible functional consequences, it is known that the LAP1 E482A mutation is located in the luminal domain [17], through which LAP1 normally interacts with and stimulates the ATPase activity of torsin family members, namely torsinA—the protein mutated in DYT1 dystonia—and torsinB [[28], [29], [30]]; as such, this mutation is predicted to have a negative impact on LAP1:torsin interaction and subsequent LAP1-induced torsin activation [31,32]. Additionally, in terms of structural implications, the LAP1 E482A mutation presumably impairs the proper folding of LAP1 [31], potentially reducing the overall stability of the mutant protein, which not only explains its diminished intracellular levels detected in patient-derived fibroblasts [17], but further suggests that the effects of this mutation could extend beyond the altered interplay between LAP1 and torsins.

Since the relevance of LAP1 in human physiology has not yet been entirely deciphered, the study of pathological mechanisms underlying LAP1-associated nuclear envelopathies, using patients' cell lines as an experimental model, is a valuable strategy to address this issue. The currently known disease-causing *TOR1AIP1* mutations, which can either affect LAP1B and LAP1C simultaneously [17,18,23,24] or solely LAP1B [13,16,[20], [21], [22]], typically culminate in depleted protein levels of the mutant LAP1 isoform(s); hence, these patients' cell lines can be regarded as human LAP1 knockdown cell models. With this in mind, the aim of our work was to investigate the repercussions of LAP1 deficiency from a broad proteomic perspective using patient-derived cells bearing the LAP1 E482A mutation. We identified differentially expressed proteins in LAP1 E482A *versus* control skin fibroblasts by liquid chromatography coupled to tandem mass spectrometry (LC–MS/MS) analysis. Using bioinformatics tools, the functional characterization of these proteins through enrichment analyses of Gene Ontology (GO) categories and signaling pathways permitted to uncover molecular mechanisms possibly deregulated in LAP1-deficient cells. To validate and complement the proteomics data, additional *in vitro* studies were performed to evaluate the deregulation of several proteins and biological processes in LAP1 E482A fibroblasts, and the consequent impact of restoring LAP1B and/or LAP1C protein levels was also assessed. Overall, our results indicate that human LAP1 depletion may have pleiotropic pathogenic effects, suggesting a putative involvement of LAP1 in novel cellular activities.

## 2 Materials and Methods

### 2.1 Antibodies

For the detection of target proteins by immunoblotting (IB) and/or immunocytochemistry (ICC), the following primary antibodies were used: mouse monoclonal anti-heat shock protein 90α family class A member 1 (HSP90α; StressMarq Biosciences (SMC-147), British Columbia, Canada; 0.5 μg/mL for IB); rabbit monoclonal anti-caldesmon 1 (Cell Signaling Technology (12503), Leiden, The Netherlands; 0.026 μg/mL for IB); mouse monoclonal anti-peroxiredoxin 6 (Santa Cruz Biotechnology (sc-166454), Heidelberg, Germany; 0.2 μg/mL for IB); mouse monoclonal anti-Fyn (Invitrogen (MA1-19331), Thermo Fisher Scientific, Waltham, Massachusetts, United States of America (USA); 1.25 μg/mL for IB); mouse monoclonal anti-histone variant H2AX phosphorylated at Ser139 (γ-H2AX; Millipore (05-636), Darmstadt, Germany; 2 μg/mL for IB); mouse monoclonal anti-puromycin (Millipore (MABE343); 0.5 μg/mL for IB); rabbit polyclonal anti-nuclear factor erythroid 2-related factor 2 (Nrf2; Invitrogen (PA5-27882); 0.41 μg/mL for IB); rabbit polyclonal anti-LAP1 (Goodchild and Dauer [33]; 0.5 μg/mL for IB); rabbit polyclonal anti-LAP1 (Atlas Antibodies (HPA050546), Bromma, Sweden; 2 μg/mL for ICC); mouse monoclonal anti-Myc tag (Cell Signaling Technology (2276); 0.32 μg/mL for ICC); and mouse monoclonal anti-hemagglutinin (HA) tag (Millipore (05-904); 5 μg/mL for ICC). Regarding the secondary antibodies, horseradish peroxidase (HRP)-linked horse anti-mouse immunoglobulin G (IgG; Cell Signaling Technology (7076); 0.015 μg/mL) and HRP-linked goat anti-rabbit IgG (Cell Signaling Technology (7074); 0.007 μg/mL) were used for immunoblotting, whereas Alexa Fluor 488-conjugated goat anti-rabbit IgG (Invitrogen (A-11008); 6.67 μg/mL) and Alexa Fluor 594-conjugated goat anti-mouse IgG (Invitrogen (A-11005); 6.67 μg/mL) were utilized for immunocytochemistry.

### 2.2 Expression vectors and DNA constructs

Human full-length LAP1B complementary DNA (cDNA) cloned into the pCMV-Myc expression vector (Clontech, Takara Bio USA, San Jose, California, USA) and human full-length LAP1C cDNA cloned into the pCMV-HA expression vector (Clontech), prepared as previously described by Santos et al. [4,34], were used in cell transfections to express Myc-LAP1B and HA-LAP1C fusion proteins, respectively (see subsection 2.4.3).

### 2.3 Human cell lines

Patient-derived skin fibroblasts bearing the LAP1 E482A missense mutation and age-/gender-matched control skin fibroblasts were used. The two fibroblast cell lines had been established from human donors, as previously reported by Dorboz et al. [17], and were made available for the present study.

### 2.4 Cell culture procedures

Human fibroblasts were cultured in Dulbecco's modified Eagle medium (DMEM; Gibco, Thermo Fisher Scientific, Waltham, Massachusetts, USA) supplemented with 15 % fetal bovine serum (FBS; Gibco) and 1 % penicillin/streptomycin (Gibco). Cells were maintained at 37 °C in a humidified atmosphere with 5 % CO_2_ and subcultured when they reached a confluency of 80–90 %.

All experiments were performed using LAP1 E482A and control fibroblasts from identical cell passages and did not exceed cell passage 15. Before starting the experiments, the fibroblast cell lines were seeded at cell-specific densities, given the different duplication rates of control and LAP1 E482A fibroblasts (≈3 and 4 days, respectively), and cultured in normal conditions during 48 h to allow them to achieve a similar confluency of 80–90 % at the beginning of the experiments.

#### 2.4.1 Cell treatments

Human fibroblasts were exposed to bleomycin (Santa Cruz Biotechnology) or hydrogen peroxide (H_2_O_2_; Sigma-Aldrich, Merck KGaA, Darmstadt, Germany) to test their susceptibility to DNA damage or oxidative stress, respectively. The two cell lines were initially seeded and grown in standard conditions for 48 h, after which cells were incubated at 37 °C in fresh culture medium with different concentrations of bleomycin or H_2_O_2_ during specific time points. A control condition in which no bleomycin or H_2_O_2_ was added to the culture medium was also included in each experiment. After cell treatments, whole cell lysates were prepared (see subsection 2.5) and analyzed by immunoblotting (see 2.10, 2.9).

• *Bleomycin treatment*

Control and LAP1 E482A fibroblasts were seeded at a density of 1 × 10^5^ cells and 1.25 × 10^5^ cells, respectively, in 6-well plates and later exposed to 25 or 50 μg/mL of bleomycin for 30 min, followed by 6 h of recovery in fresh culture medium.

• H_2_O_2_ treatment

Control and LAP1 E482A fibroblasts were seeded at a density of 7.5 × 10^4^ cells and 9 × 10^4^ cells, respectively, in 6-well plates and then treated with 100 or 200 μM of H_2_O_2_ during 24 h.

#### 2.4.2 Analysis of global protein synthesis

To assess the overall rate of translation, human fibroblasts were cultured in the presence of puromycin (Sigma-Aldrich). After seeding control and LAP1 E482A fibroblasts at a density of 1 × 10^5^ cells and 1.25 × 10^5^ cells, respectively, in 35 mm culture dishes and allowing them to standardly grow for 48 h, cells were incubated at 37 °C in fresh culture medium containing 10 μg/mL of puromycin during 15 min. Afterwards, cells were harvested (see subsection 2.5) for posterior immunoblotting analysis (see 2.10, 2.9).

#### 2.4.3 Cell transfection

Transient transfections of human fibroblasts were performed to rescue LAP1 isoforms' protein expression in patient-derived LAP1-deficient cells. Control and LAP1 E482A fibroblasts, seeded at a density of 1 × 10^5^ cells and 1.25 × 10^5^ cells, respectively, in 6-well plates (with or without glass coverslips), were cultured in standard conditions for 48 h. After that period, cells were transfected with LAP1B- and/or LAP1C-encoding DNA constructs or corresponding empty expression vectors (see subsection 2.2) for 24 h using the TurboFect transfection reagent (Thermo Scientific, Thermo Fisher Scientific, Waltham, Massachusetts, USA), following the manufacturer's protocol with some modifications. Briefly, DNA diluted in DMEM was incubated with TurboFect at a DNA:TurboFect ratio of 1:2 during 20 min, after which DNA:TurboFect complexes were added to cells. Upon 6 h of incubation at 37 °C, the culture medium was replaced and cells were again incubated at 37 °C until reaching a total of 24 h of transfection. LAP1 E482A fibroblasts were transfected with 1 μg of Myc-LAP1B, 0.5 μg of HA-LAP1C or 0.5 μg of each LAP1-encoding DNA construct to restore the protein levels of LAP1B, LAP1C or both LAP1 isoforms, respectively. As control conditions for each transfection experiment, LAP1 E482A and control fibroblasts were transfected with 1 μg of pCMV-Myc, 0.5 μg of pCMV-HA or 0.5 μg of each expression vector, respectively. After transfection, the preparation of whole cell lysates (see subsection 2.5) and subsequent immunoblotting analysis (see 2.10, 2.9) were carried out or, alternatively, cells were fixed and analyzed by immunocytochemistry (see subsection 2.12).

### 2.5 Preparation of whole cell lysates

For the LC–MS/MS analysis of human fibroblasts' total protein extracts, cells in confluent 60 mm culture dishes, which had been grown in standard conditions, were scraped into urea lysis buffer (6 M urea; 200 mM ammonium bicarbonate). Following an incubation period of 30 min, whole cell lysates were sonicated thrice for 5 s (0.5 cycles, 60 % amplitude) and centrifuged at 20,000 g for 15 min. Supernatants (total protein extracts) were collected and total protein concentration was measured using the Pierce bicinchoninic acid (BCA) protein assay kit (Thermo Scientific), according to the manufacturer's instructions. After adjusting the concentration of protein extracts to 1 μg/μL through the addition of urea lysis buffer, 10 μg of each sample were reduced with dithiothreitol (DTT; 30 nmol) at 37 °C for 1 h and alkylated with iodoacetamide (60 nmol) at 25 °C for 30 min in the dark. Subsequently, protein extracts were diluted to 2 M urea with 200 mM ammonium bicarbonate and digested with endoproteinase LysC (FUJIFILM Wako Pure Chemical Corporation, Osaka, Japan; 1:10 *w/w*) at 37 °C for 6 h, after which were diluted 2-fold with 200 mM ammonium bicarbonate and digested with trypsin (Promega, Madison, Wisconsin, USA; 1:10 *w/w*) at 37 °C overnight. The resulting peptide mixes were acidified with formic acid and desalted using a MicroSpin C18 column (The Nest Group, Ipswich, Massachusetts, USA), followed by LC–MS/MS analysis (see subsection 2.7).

For the immunoblotting analysis of total protein extracts obtained from human fibroblasts under basal conditions, cells in confluent T75 culture flasks were lysed in boiling 1 % sodium dodecyl sulfate (SDS). Similarly, upon incubation with bleomycin, H_2_O_2_ (see subsection 2.4.1) or puromycin (see subsection 2.4.2), or transfection with DNA constructs (see subsection 2.4.3), human fibroblasts were lysed by resuspension in boiling 1 % SDS. Next, whole cell lysates were boiled at 90 °C for 10 min and sonicated for 10 s (0.5 cycles, 60 % amplitude). Total protein concentration was determined using the Pierce BCA protein assay kit and samples were posteriorly separated by sodium dodecyl sulfate–polyacrylamide gel electrophoresis (SDS–PAGE; see subsection 2.9) and analyzed by immunoblotting (see subsection 2.10).

### 2.6 Extraction of detergent-insoluble protein fractions

For the extraction of detergent-insoluble proteins from human fibroblasts in a basal state, cells in confluent 60 mm culture dishes were firstly harvested through trypsinization and cell pellets obtained by centrifugation were then resuspended in ice-cold protein lysis buffer (0.5 % Triton X-100; 50 mM 4-(2-hydroxyethyl)-1-piperazineethanesulfonic acid (HEPES), pH = 7; 250 mM sodium chloride (NaCl); 1 mM DTT; 2 mM ethylenediaminetetraacetic acid (EDTA); 1 mM ethylene glycol-bis(β-aminoethyl ether)-N,N,N′,N′-tetraacetic acid (EGTA)) supplemented with phosphatase and protease inhibitors (1 mM sodium fluoride (NaF); 1 mM sodium orthovanadate (Na_3_VO_4_); 1 mM phenylmethylsulfonyl fluoride (PMSF); 1 × cOmplete, EDTA-free protease inhibitor cocktail (Roche, Basel, Switzerland)). Whole cell lysates were sonicated twice for 15 s (0.5 cycles, 60 % amplitude) and centrifuged at 200 g for 20 min at 4 °C. Supernatants (total protein extracts) were collected and total protein concentration was assessed using the Pierce BCA protein assay kit. To obtain detergent-insoluble protein fractions, 200 μg of total protein extracts were used in subsequent steps. After centrifuging these samples at 16,000 g for 20 min at 4 °C, pellets were solubilized in ice-cold protein lysis buffer containing 2 % NP-40 and sonicated for 20 s (0.5 cycles, 60 % amplitude), which was followed by an additional centrifugation at 16,000 g for 20 min at 4 °C. The resulting pellets (insoluble protein fractions) were resuspended in ice-cold protein lysis buffer and sonicated for 20 s (0.5 cycles, 60 % amplitude), being subsequently subjected to analysis through SDS–PAGE (see subsection 2.9) and polyacrylamide gel staining (see subsection 2.11).

### 2.7 LC–MS/MS

Three replicate samples obtained from each fibroblast cell line (see subsection 2.5) were analyzed using an Orbitrap Fusion Lumos mass spectrometer (Thermo Scientific, Thermo Fisher Scientific, San Jose, California, USA) coupled to an EASY-nLC 1200 nanoflow liquid chromatography system (Proxeon, Thermo Fisher Scientific, Odense, Denmark). Peptides were loaded directly onto the analytical column and separated by reversed-phase chromatography using a 50 cm column with an inner diameter of 75 μm, packed with 2 μm C18 particles spectrometer (Thermo Scientific). Chromatographic gradients started at 95 % buffer A (0.1 % formic acid in water)/5 % buffer B (0.1 % formic acid in 80 % acetonitrile) with a flow rate of 300 nL/min for 5 min and gradually increased to 75 % buffer A/25 % buffer B in 79 min and then to 60 % buffer A/40 % buffer B in 11 min. After each analysis, the column was washed with 10 % buffer A/90 % buffer B for 10 min.

The mass spectrometer was operated in the positive ionization mode, with the nano-spray voltage set at 2.4 kV and the source temperature at 305 °C. The acquisition was performed in the data-dependent acquisition (DDA) mode and full mass spectrometry (MS) scans, with 1 microscan at a resolution of 120,000, were used over an *m/z* range of 350–1400, with detection in the Orbitrap mass analyzer. The auto gain control (AGC) was set to ‘standard’ and the injection time to ‘auto’. In each cycle of DDA analysis, following each survey scan, the most intense ions above a threshold ion count of 10,000 were selected for fragmentation. The number of selected precursor ions for fragmentation was determined by the ‘Top Speed’ acquisition algorithm and a dynamic exclusion of 60 s. Fragment ion spectra were produced via high-energy collision dissociation (HCD) at normalized collision energy of 28 % and acquired in the Ion Trap mass analyzer. The AGC was set to 2e4 and an isolation window of 0.7 *m/z* as well as a maximum injection time of 12 ms were used. Digested bovine serum albumin (BSA) MS standard (New England Biolabs, Ipswich, Massachusetts, USA) was analyzed between each sample to avoid sample carryover and to assure stability of the instrument. The QCloud quality control system [35] was used to monitor the instrument's longitudinal performance during the LC–MS/MS experiments.

Acquired spectra were analyzed using the Proteome Discoverer software suite (version 2.5; Thermo Scientific) and the Mascot search engine (version 2.6; Matrix Science, London, United Kingdom (UK) [36]). LC–MS/MS data were searched against a Swiss-Prot human database (as in January 2021, 20,395 entries) plus a list of common contaminants [37] and all the corresponding decoy entries. For peptide identification, a precursor ion mass tolerance of 7 ppm was used for MS1 level, trypsin was chosen as enzyme and up to three missed cleavages were allowed. The fragment ion mass tolerance was set to 0.5 Da for MS2 spectra. Oxidation of methionine and N-terminal protein acetylation were used as variable modifications, whereas carbamidomethylation on cysteines was set as a fixed modification. The false discovery rate (FDR) in peptide identification was set to a maximum of 5 %. Peptide quantification data were retrieved from the ‘Precursor ions quantifier’ node from Proteome Discoverer software (version 2.5) using a mass tolerance of 2 ppm for the peptide extracted ion current (XIC). The obtained values were normalized by protein abundance and used to calculate the protein fold change (log2 of the ratio between protein abundance in LAP1 E482A fibroblasts and in control fibroblasts), *p*-value and adjusted *p*-value. The MS proteomics data have been deposited to the ProteomeXchange consortium [38] via the Proteomics Identifications (PRIDE) [39] partner repository with the dataset identifier PXD035200.

### 2.8 Bioinformatic analysis of LC–MS/MS data

Before starting the *in silico* analysis, several criteria were used to manually select the relevant differentially expressed human proteins from the full list of 7274 proteins detected in the LC–MS/MS experiments. First, proteins exhibiting altered abundance levels in LAP1 E482A fibroblasts relatively to control fibroblasts, and for which an adjusted *p*-value < 0.1 was obtained, were selected. Based on this criterion, 131 proteins found in two or three replicates of LAP1 E482A and control samples were identified; among these, there was a contaminant bovine protein that was excluded. Second, 148 proteins exclusive of control fibroblasts (i.e. found in two or three replicates of control samples and in none of LAP1 E482A samples) were also selected. Third, 121 proteins exclusive of LAP1 E482A fibroblasts (i.e. found in two or three replicates of LAP1 E482A samples and in none of control samples) were additionally selected. Fourth, from the 399 proteins identified above, only those presenting a fold change higher than 0.5 or lower than −0.5 were considered for further analysis, which led to the exclusion of 13 proteins. Hence, 386 differentially expressed proteins were selected for the bioinformatic analysis. An initial characterization focused on protein class categories was performed using the Protein ANalysis THrough Evolutionary Relationships (PANTHER) online resource (version 17.0; accessed on March 2022) [40]. Moreover, a functional enrichment analysis of GO biological process, GO molecular function, GO cellular component, Kyoto Encyclopedia of Genes and Genomes (KEGG) pathways and Reactome pathways annotations was carried out employing the STRING database (version 11.5; accessed on March 2022) [41]. The human whole genome was used as a reference list for the statistical analysis of enriched terms.

Besides this global bioinformatic analysis covering all differentially expressed proteins, a more specific, cluster-centered analysis was also conducted. Using the STRING database [41], the differentially expressed proteins were firstly organized into a general protein–protein interaction (PPI) network, wherein only physical protein associations were considered for the construction of this network, which was achieved by selecting the physical network subtype (experiments, databases and text mining as active interaction sources; minimum interaction score of 0.4). Next, the proteins connected by physical associations in the original PPI network were grouped into clusters by applying the Markov clustering (MCL) algorithm (inflation parameter of 1.8). Similar functional enrichment analyses of GO categories and biological pathways were performed for each protein cluster. For the graphical representation of the PPI networks retrieved from the STRING database [41] (i.e. general PPI network including all differentially expressed proteins and PPI subnetworks corresponding to protein clusters), the Cytoscape software (version 3.9.1) [42] was used.

### 2.9 SDS–PAGE

Upon preparation of whole cell lysates (see subsection 2.5) or detergent-insoluble protein fractions (see subsection 2.6), samples were boiled in 1 × loading buffer (62.5 mM Tris, pH = 6.8; 2 % SDS; 10 % glycerol; 5 % β-mercaptoethanol; 0.0025 % bromophenol blue) at 90 °C for 10 min prior to loading into polyacrylamide gels. Total protein extracts obtained from human fibroblasts grown in basal conditions, treated with bleomycin or H_2_O_2_, or transfected with DNA constructs were separated on a gradient (5–20 %) polyacrylamide gel, whereas those collected from cells incubated with puromycin were resolved on a 10 % polyacrylamide gel. Following SDS–PAGE, proteins were electrophoretically transferred onto nitrocellulose membranes (0.2 μm pore size; GE Healthcare, Buckinghamshire, UK) and their immunological detection with specific antibodies was posteriorly carried out (see subsection 2.10). Alternatively, to analyze the insoluble/total protein profile of human fibroblasts in a basal state, insoluble protein fractions and corresponding total protein extracts were separated on a gradient polyacrylamide gel, which was later subjected to staining (see subsection 2.11).

### 2.10 Immunoblotting

To assess the total protein amount loaded in each sample, nitrocellulose membranes were initially stained with Ponceau S solution (5 % acetic acid; 0.1 % Ponceau S) for 10 min. For immunoblotting, membranes were blocked in 5 % BSA/1 × Tris-buffered saline with 0.1 % Tween-20 (TBS-T) for 3 h and then incubated with primary antibodies (see subsection 2.1) in 3 % BSA/1 × TBS-T for 2 h, followed by overnight incubation at 4 °C. Afterwards, membranes were incubated with HRP-conjugated secondary antibodies (see subsection 2.1) in 5 % fat-free dry milk/1 × TBS-T for 1 h. Protein bands were visualized in a ChemiDoc imaging system (Bio-Rad, Hercules, California, USA) by enhanced chemiluminescence (ECL) detection. The Image Lab software (Bio-Rad) was utilized for the quantitative analysis of HSP90α, caldesmon 1, peroxiredoxin 6, Fyn, γ-H2AX, Nrf2 and LAP1's protein levels as well as puromycin incorporation into nascent proteins, with Ponceau S staining being used as a protein loading control for data normalization.

### 2.11 Polyacrylamide gel staining

Polyacrylamide gels were stained with BlueSafe solution (NZYTech, Lisbon, Portugal) for 1 h and scanned in a GS-800 imaging densitometer (Bio-Rad) to detect stained protein bands. Quantification of insoluble/total protein ratio was achieved using the Image Lab software (Bio-Rad).

### 2.12 Immunocytochemistry and confocal microphotograph acquisition

Upon transfection of DNA constructs, human fibroblasts were incubated with 3.7 % paraformaldehyde (PFA) during 20 min for cell fixation. After being permeabilized using 0.2 % Triton X-100/1 × phosphate buffered saline (PBS) for 10 min, cells were blocked in 3 % BSA/1 × PBS for 2 h. This was followed by incubation with primary antibodies against LAP1, Myc tag and/or HA tag (see subsection 2.1) in 3 % BSA/1 × PBS for 2 h, after which cells were incubated with Alexa Fluor 488- and/or Alexa Fluor 594-conjugated secondary antibodies (see subsection 2.1) in 3 % BSA/1 × PBS for 1 h in the dark. Coverslips were then mounted on microscope slides using 4’,6-diamidino-2-phenylindole (DAPI)-containing Vectashield anti-fade mounting medium (Vector Laboratories, Burlingame, California, USA). To visualize the immunocytochemistry preparations, an LSM 880 confocal laser scanning microscope with Airyscan (Zeiss, Jena, Germany) and a plan-apochromat 63 ×/1.4 oil immersion DIC M27 objective were used. Fluorescence excitation was achieved by employing the 405 nm (DAPI), 488 nm (Alexa Fluor 488) and 561 nm (Alexa Fluor 594) laser lines, and the fluorescent signals were acquired in the range of 410–495 nm (DAPI), 495–584 nm (Alexa Fluor 488) and 585–733 nm (Alexa Fluor 594). The acquisition of microphotographs (image size of 512 × 512 pixels with a pixel size of 0.26 μm) was performed in multiple optical sections in the Z-axis to permit the production of z-stacks (≈10–15 slices with a z-step size of 0.37 μm).

### 2.13 Statistical analysis

For the functional enrichment analyses of differentially expressed proteins using the STRING database [41], the Benjamini–Hochberg procedure was employed to control the FDR for multiple testing within each category. Enriched terms were identified as those for which a *p*-value < 0.05 was obtained.

Regarding the quantitative results of experiments involving immunoblotting and polyacrylamide gel staining, data were expressed as mean ± standard error of the mean (SEM) of, at least, three independent samples. The statistical analysis was carried out using the GraphPad Prism 9 software (GraphPad Software, San Diego, California, USA) and is specified in figure captions. The parametric unpaired *t*-test was applied to compare relative levels of target proteins, puromycin incorporation into nascent peptides and insoluble/total protein ratio between LAP1 E482A and control fibroblasts under the same experimental condition. For the comparison of relative protein levels between untreated and several treatment groups for each fibroblast cell line, the parametric one-way Analysis of Variance (ANOVA) test or the non-parametric Kruskal–Wallis test was used, followed by the Dunnett's/Tukey's or Dunn's multiple comparisons test, respectively. Values of *p* < 0.05 were considered statistically significant.

## 3 Results

With the objective of elucidating the physiological relevance of LAP1 and the pathological effects of its deficiency in human cells, we used different experimental approaches, from LC–MS/MS to *in vitro* functional assays, to identify deregulated proteins and biological processes in patient-derived fibroblasts carrying the LAP1 E482A missense mutation, which has been shown to result in strongly reduced LAP1 protein levels [17]. A summarized view of the experimental design of our work is depicted in Fig. 1.

**Fig. 1:**
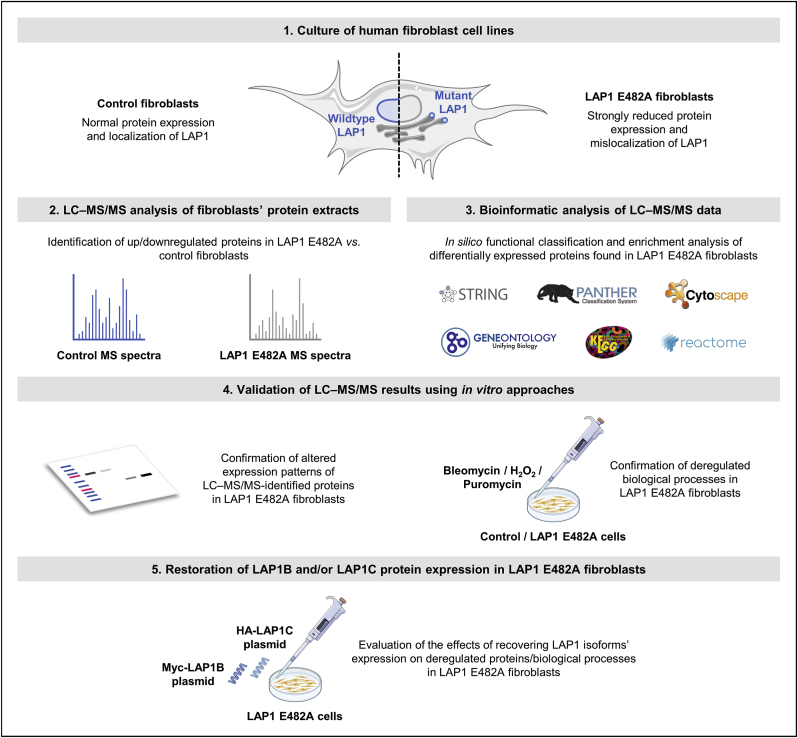
Schematic representation of the experimental design of this study. H_2_O_2_, hydrogen peroxide; HA, hemagglutinin; LAP1, lamina-associated polypeptide 1; LC–MS/MS, liquid chromatography coupled to tandem mass spectrometry; MS, mass spectrometry.

### 3.1 Global functional characterization of differentially expressed proteins, identified by LC–MS/MS, in LAP1-deficient human fibroblasts through *in silico* analysis

**Fig. 2:**
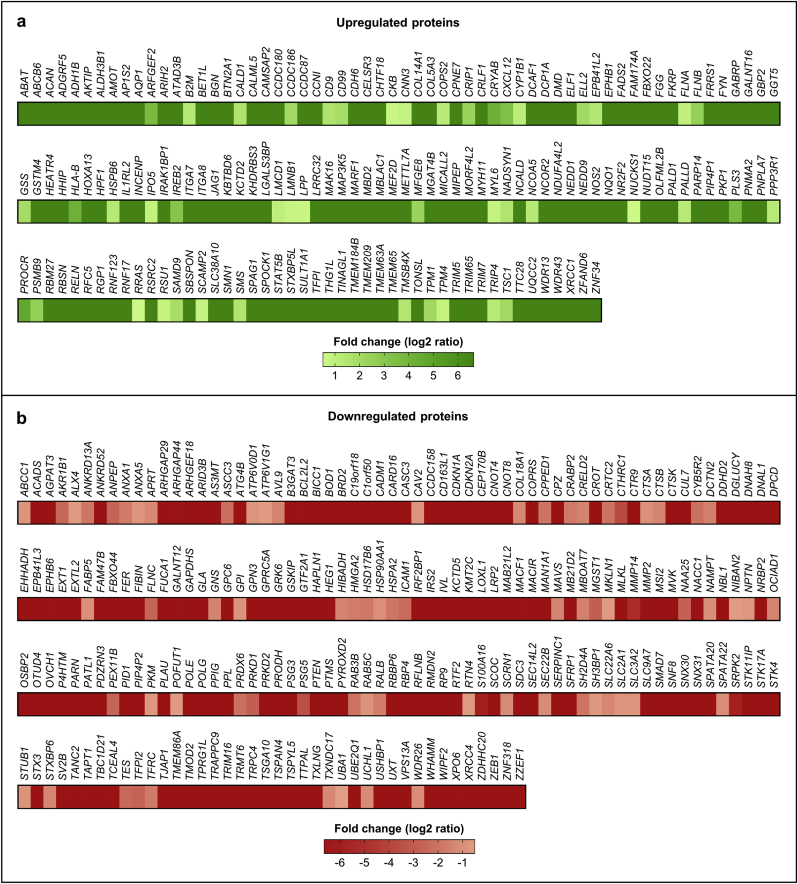
Heat map of differentially expressed proteins found in LAP1 E482A fibroblasts as compared to control fibroblasts. The protein fold change (log2 of the ratio between protein abundance in LAP1 E482A fibroblasts and in control fibroblasts) of **(a)** upregulated (0.5 < log2 ratio ≤ 6.64) and **(b)** downregulated (−6.64 ≤ log2 ratio < −0.5) proteins (identified by gene name) detected by LC–MS/MS is shown. The heat map was created using the GraphPad Prism 9 software. LAP1, lamina-associated polypeptide 1; LC–MS/MS, liquid chromatography coupled to tandem mass spectrometry.

Firstly, we performed a quantitative and functional proteome analysis to uncover distinguishing features in the global protein expression profile of LAP1 E482A *versus* control fibroblasts. Whole cell lysates obtained in steady-state conditions were analyzed by LC–MS/MS and the entire dataset of identified proteins was then processed to select those exhibiting significantly altered abundance levels between the two cell lines, as detailed in the Materials and Methods section. After this analysis, 386 differentially expressed proteins were found in LAP1 E482A fibroblasts relatively to control fibroblasts (Fig. 2), more precisely 166 upregulated proteins (Fig. 2a; Supplementary Table S1) and 220 downregulated proteins (Fig. 2b; Supplementary Table S2). Of note, it should be mentioned that a considerable decrease in the protein abundance of LAP1 was detected, as expected, in LAP1 E482A fibroblasts when compared to control fibroblasts; however, since the corresponding adjusted *p*-value was slightly higher than 0.1, LAP1 was excluded from the final list of differentially expressed proteins identified by LC–MS/MS (see the selection criteria in subsection 2.8).

**Fig. 3:**
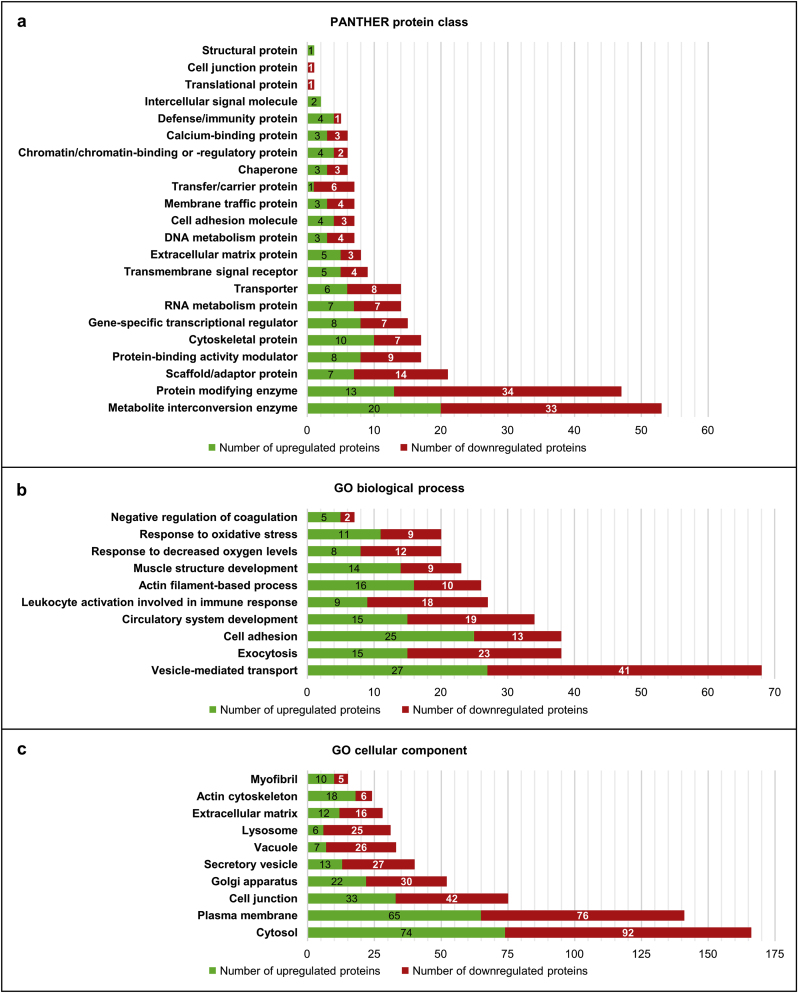
Functional characterization of differentially expressed proteins found in LAP1 E482A fibroblasts as compared to control fibroblasts. **(a)** Functional classification based on PANTHER protein class categories. From a total of 386 proteins under analysis, 271 protein class hits distributed across 22 main categories were retrieved using the PANTHER online resource [40]. **(b, c)** Functional enrichment analysis of **(b)** GO biological process and **(c)** GO cellular component categories. Some enriched GO terms (summarizing the broad spectrum of over-represented annotations) retrieved from the STRING database [41] are shown. Green and red bars represent, respectively, the number of upregulated and downregulated proteins that were annotated with each protein class category or GO term. DNA, deoxyribonucleic acid; GO, Gene Ontology; LAP1, lamina-associated polypeptide 1; PANTHER, Protein ANalysis THrough Evolutionary Relationships; RNA, ribonucleic acid. (For interpretation of the references to colour in this figure legend, the reader is referred to the Web version of this article.)

Following their identification, the deregulated proteins detected in LAP1 E482A fibroblasts were subjected to *in silico* functional analyses (Fig. 3). The Protein ANalysis THrough Evolutionary Relationships (PANTHER) online resource [40] was initially used to characterize the protein classes to which they belong. After examining all differentially expressed proteins, PANTHER protein class annotations distributed across 22 main categories were retrieved for 271 of them (Fig. 3a; Supplementary Table S3). The results revealed that a large number of these proteins are metabolite interconversion enzymes (e.g. transferases and oxidoreductases) and protein modifying enzymes (e.g. proteases, protein kinases and phosphatases, and ubiquitin–protein ligases) (Fig. 3a; Supplementary Table S3). A smaller portion of the mapped proteins comprises scaffold/adaptor proteins, protein-binding activity modulators (e.g. G proteins and kinase inhibitors/activators), cytoskeletal proteins (e.g. actin-, microtubule- and intermediate filament-binding proteins), gene-specific transcriptional regulators (e.g. DNA-binding transcription factors and transcription cofactors), RNA metabolism proteins (e.g. RNA splicing factors and general transcription factors) and transporters (e.g. ion channels, primary active transporters and secondary carriers) (Fig. 3a; Supplementary Table S3). Other represented protein class categories include, for instance, transmembrane signal receptors, cell adhesion molecules, chaperones and defense/immunity proteins (Fig. 3a; Supplementary Table S3).

Subsequently, a functional enrichment analysis of GO categories and biological pathways was carried out using the STRING database [41] to further characterize the deregulated proteins identified in LAP1 E482A fibroblasts (Fig. 3b and c; Supplementary Table S4). The over-represented GO biological process annotations evidence that they exhibit diverse physiological roles, such as vesicle-mediated transport, exocytosis, cell adhesion, circulatory system development, leukocyte activation involved in immune response, muscle structure development and response to oxidative stress (Fig. 3b; Supplementary Table S4). Moreover, the enriched terms of GO cellular component demonstrate that these proteins are widely distributed in the cell, being localized, for example, in the cytosol, plasma membrane, cell junctions, Golgi apparatus, secretory vesicles, lysosomes, extracellular matrix (ECM) and actin cytoskeleton (Fig. 3c; Supplementary Table S4).

Taken together, these findings suggest that LAP1 depletion may have a tremendous impact on the global functioning of human cells by affecting numerous proteins that fulfill a broad range of biological activities in distinct subcellular compartments. Noteworthy, the protein–protein interaction (PPI) network comprising all differentially expressed proteins uncovered in LAP1 E482A fibroblasts, which was obtained using the STRING database [41], shows the existence of several subgroups of interacting proteins (Supplementary Fig. S1). We hypothesized that a particular function/signaling pathway could be assigned to a subset of proteins connected by physical associations (and, hence, more prone to be functionally related to each other), giving more information about potential pathological mechanisms underlying LAP1-associated diseases than the previous generalized approach. Thus, a more specific *in silico* functional analysis centered on protein clusters was performed next.

### 3.2 Cluster-specific functional characterization of differentially expressed proteins, identified by LC–MS/MS, in LAP1-deficient human fibroblasts through *in silico* analysis

**Fig. 4:**
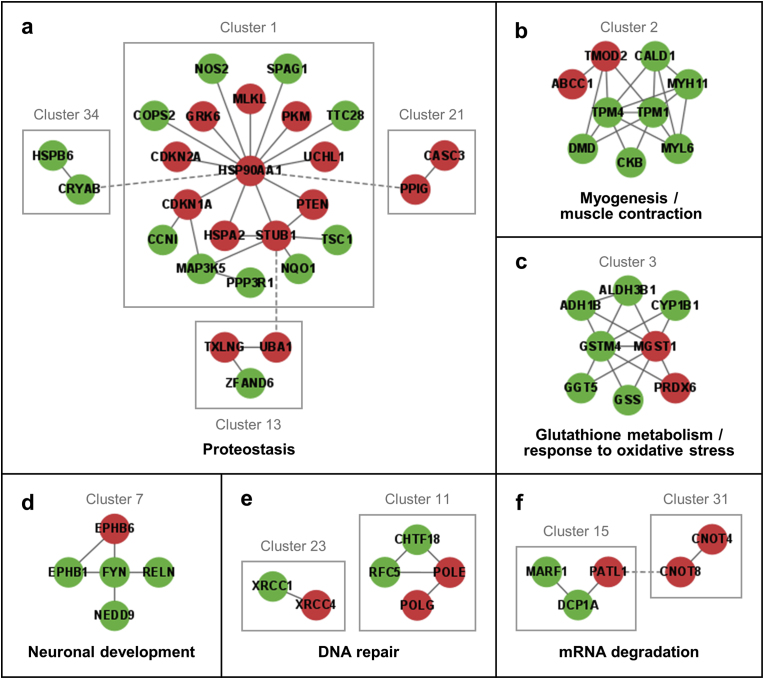
PPI subnetworks of **(a)** clusters 1, 13, 21 and 34 (proteostasis), **(b)** cluster 2 (myogenesis/muscle contraction), **(c)** cluster 3 (glutathione metabolism/response to oxidative stress), **(d)** cluster 7 (neuronal development), **(e)** clusters 11 and 23 (DNA repair), and **(f)** clusters 15 and 31 (mRNA degradation) comprising differentially expressed proteins found in LAP1 E482A fibroblasts as compared to control fibroblasts. The PPI data were retrieved from the STRING database [41] and the PPI subnetworks constructed using the Cytoscape software [42]. Green and red nodes represent, respectively, upregulated and downregulated proteins (identified by gene name), while edges denote physical associations between proteins (solid edges for interactions between proteins of the same cluster and dashed edges for interactions between proteins of different clusters). DNA, deoxyribonucleic acid; LAP1, lamina-associated polypeptide 1; mRNA, messenger RNA; PPI, protein–protein interaction; RNA, ribonucleic acid. (For interpretation of the references to colour in this figure legend, the reader is referred to the Web version of this article.)

In order to split the main PPI network (Supplementary Fig. S1) into clusters, the Markov clustering (MCL) algorithm of the STRING database [41] was employed. From a total of 386 differentially expressed proteins detected in LAP1 E482A fibroblasts, 145 proteins connected by physical interactions were grouped into 41 clusters (Supplementary Fig. S2; Supplementary Table S5). Using the STRING database [41], each cluster was characterized from a functional point of view through an enrichment analysis of GO categories and biological pathways. Of note, given that some proteins of clusters 13, 21 and 34 were both physically (Fig. 4a) and functionally (i.e. belong to the same protein class; Supplementary Table S3) associated to proteins of cluster 1, these four clusters were analyzed together; the same applies to clusters 15 and 31 (Fig. 4f; Supplementary Table S3). Over-represented terms of GO biological process, GO molecular function, GO cellular component, Kyoto Encyclopedia of Genes and Genomes (KEGG) pathways and/or Reactome pathways were retrieved for most clusters and are listed in Supplementary Tables S6–S35. This cluster-based functional characterization of the deregulated proteins found in LAP1 E482A fibroblasts permitted to correlate subgroups of interacting proteins with specific physiological processes/signaling cascades, including proteostasis, myogenesis/muscle contraction, glutathione metabolism/response to oxidative stress, intracellular trafficking, neuronal development, ECM organization, DNA repair, nucleocytoplasmic transport, messenger RNA (mRNA) degradation, cytoskeleton organization and adaptive immune response (Supplementary Tables S6–S35). The results of the bioinformatic analysis of some of these clusters (Fig. 4), which provide clues about potential consequences of LAP1 deficiency, are briefly described below; these include: (i) clusters associated with more universal (i.e. non-cell-specific) cellular activities that are possibly altered in multiple cell types lacking a normal LAP1 protein expression (e.g. proteostasis (Fig. 4a), glutathione metabolism/oxidative stress response (Fig. 4c), DNA repair (Fig. 4e) and mRNA decay (Fig. 4f)); and (ii) clusters associated with tissue-specific developmental processes (e.g. myogenesis/muscle contraction (Fig. 4b) and neurogenesis (Fig. 4d)) that are more closely related to the muscular and neurological disorders linked to *TOR1AIP1* mutations, inclusively the LAP1 E482A mutation.

#### 3.2.1 Proteostasis

The PPI subnetwork combining clusters 1, 13, 21 and 34 (Fig. 4a) is enriched with key modulators of proteostasis that are implicated in protein folding and stabilization, cellular response to stress, regulation of protein ubiquitination, chaperone-mediated autophagy and aging (Supplementary Table S6). It should also be highlighted the association of these proteins with several relevant signaling events/pathways, namely heat shock factor 1 (HSF1)-dependent transactivation linked to heat shock response, protein processing in the ER, phosphatidylinositol 3-kinase (PI3K)-Akt signaling pathway involved in the control of protein synthesis and other cellular activities, and p53 signaling pathway induced by cellular stress (Supplementary Table S6).

#### 3.2.2 Myogenesis and muscle contraction

Cluster 2's PPI subnetwork (Fig. 4b) contains many proteins that regulate certain aspects of muscle development and contraction processes, such as myofibril assembly, actin cytoskeleton organization and muscle filament sliding (Supplementary Table S7). In addition, these proteins appear to be associated with the pathophysiology of hypertrophic and dilated cardiomyopathies (Supplementary Table S7).

#### 3.2.3 Glutathione metabolism and response to oxidative stress

The *in silico* analysis of cluster 3's PPI subnetwork (Fig. 4c) unveiled an enrichment of various terms correlated with the major intracellular antioxidant glutathione, among which: glutathione synthesis and recycling, cellular response to oxidative stress, glutathione conjugation, response to xenobiotic stimulus as well as glutathione peroxidase and transferase activities (Supplementary Table S8).

#### 3.2.4 Neuronal development

The PPI subnetwork of cluster 7 (Fig. 4d) shows a strong association with neurogenesis, given the involvement of its proteins in cell migration, dendrite morphogenesis, axon guidance, regulation of synapse organization and modulation of chemical synaptic transmission (Supplementary Table S12). Furthermore, they mediate two important signaling cascades required for neuronal development and maturation, more specifically the ephrin and reelin signaling pathways (Supplementary Table S12).

#### 3.2.5 DNA repair

The bioinformatic analyses of the two independent PPI subnetworks of clusters 11 and 23 (Fig. 4e) revealed that both are functionally related to DNA repair (Supplementary Tables S16 and S24, respectively). Regarding the proteins of cluster 11, these participate, for example, in the biological processes of DNA synthesis involved in DNA repair and DNA replication, as well as in distinct DNA repair signaling cascades, including homologous recombination repair (HRR), nucleotide excision repair (NER) and base excision repair (BER) (Supplementary Table S16). In turn, the proteins of cluster 23 seem to regulate DNA ligation involved in DNA repair and the non-homologous end joining (NHEJ) signaling pathway (Supplementary Table S24).

#### 3.2.6 mRNA degradation

The proteins of the PPI subnetwork linking clusters 15 and 31 (Fig. 4f) modulate RNA degradation, in particular deadenylation-dependent mRNA decay, being implicated in nuclear-transcribed mRNA poly(A) tail shortening, deadenylation-dependent decapping of nuclear-transcribed mRNA, exonucleolytic catabolism of deadenylated mRNA and post-transcriptional gene silencing (Supplementary Table S18).

In essence, considering the results of the bioinformatic analysis centered on clusters of differentially expressed proteins detected in LAP1 E482A fibroblasts, it appears that LAP1 depletion may culminate in the deregulation of DNA repair pathways (e.g. NHEJ and BER), mRNA decay via deadenylation-dependent mechanisms, protein folding/degradation, glutathione metabolism/antioxidant response, as well as neuronal and muscle development. Additional functional studies will be required to clarify the exact alterations occurring in these physiological processes in LAP1-deficient cells/tissues, with some insights being provided in subsection 3.4.

### 3.3 Validation of differentially expressed proteins, identified by LC–MS/MS, in LAP1-deficient human fibroblasts through immunoblotting analysis

**Fig. 5:**
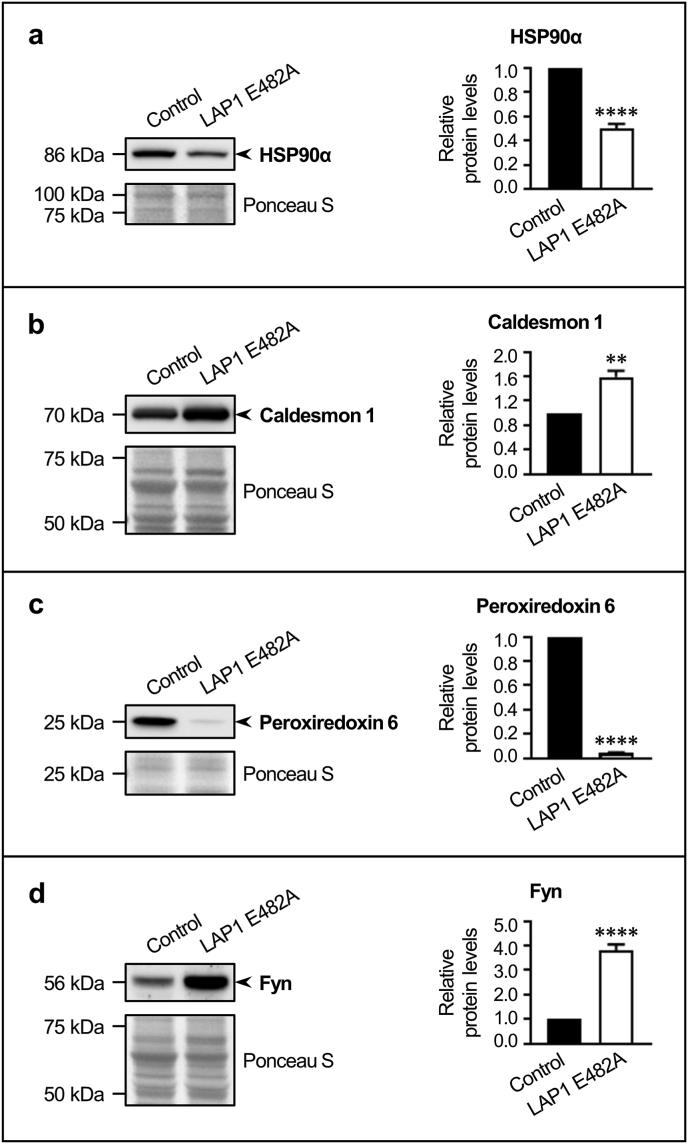
Validation of alterations in the protein levels of several LC–MS/MS-identified proteins between LAP1 E482A fibroblasts and control fibroblasts. Relative protein levels of **(a)** HSP90α, **(b)** caldesmon 1, **(c)** peroxiredoxin 6 and **(d)** Fyn in LAP1 E482A fibroblasts (estimated in relation to control fibroblasts). Whole cell lysates were analyzed by immunoblotting using specific antibodies against the human target proteins; a representative blot is shown for each protein. Quantitative data are presented as mean ± SEM (*n* = 6). Ponceau S staining was used as protein loading control for data normalization before determining relative protein levels. **p < 0.01 and ****p < 0.0001 for comparisons between control and LAP1 E482A fibroblasts using the unpaired *t*-test. HSP90α, heat shock protein 90α family class A member 1; LAP1, lamina-associated polypeptide 1; LC–MS/MS, liquid chromatography coupled to tandem mass spectrometry; SEM, standard error of the mean.

To corroborate the LC–MS/MS data by employing an alternative experimental approach, we analyzed by immunoblotting a subset of proteins for which altered abundance levels were detected in LAP1 E482A *versus* control fibroblasts (Fig. 5). Total protein extracts collected from both cell lines in a basal state were used for the immunological detection of four proteins selected for additional validation: (i) heat shock protein (HSP) 90α family class A member 1 (HSP90α), a molecular chaperone (Fig. 5a); (ii) caldesmon 1, an actin-binding cytoskeletal protein (Fig. 5b); (iii) peroxiredoxin 6, a peroxidase enzyme (Fig. 5c); and (iv) Fyn, a protein kinase (Fig. 5d). In agreement with the LC–MS/MS findings, the immunoblotting results confirmed that the protein levels of HSP90α (Fig. 5a) and peroxiredoxin 6 (Fig. 5c) were significantly decreased, while those of caldesmon 1 (Fig. 5b) and Fyn (Fig. 5d) were significantly increased, in LAP1 E482A fibroblasts comparatively to control fibroblasts.

### 3.4 Validation of deregulated biological processes, identified by *in silico* analysis of LC–MS/MS data, in LAP1-deficient human fibroblasts through *in vitro* functional assays

**Fig. 6:**
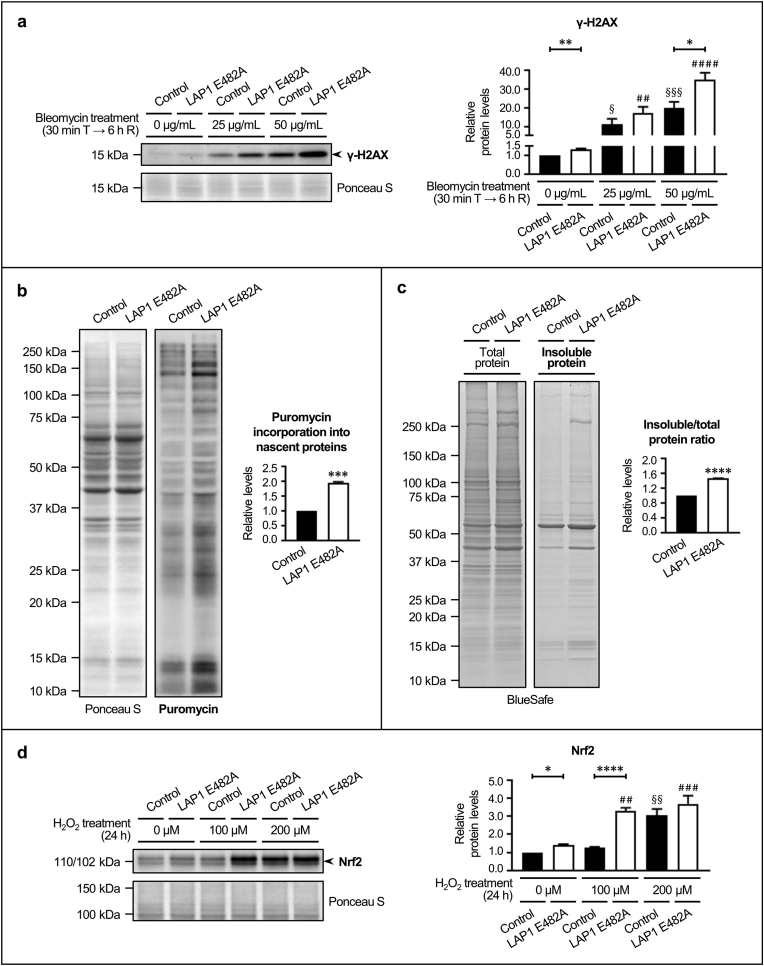
Validation of alterations in the biological processes of **(a)** DDR/DNA repair, **(b)** translation, **(c)** proteostasis and **(d)** oxidative stress response between LAP1 E482A fibroblasts and control fibroblasts. **(a)** Relative γ-H2AX levels at baseline (0 μg/mL) and upon cell exposure to 25 and 50 μg/mL of bleomycin for 30 min (T), followed by 6 h of recovery (R), in control and LAP1 E482A fibroblasts (estimated in relation to untreated control fibroblasts). After bleomycin treatment, whole cell lysates were analyzed by immunoblotting using a specific antibody against γ-H2AX to measure DNA damage levels; a representative blot is shown. **(b)** Relative levels of puromycin incorporation into nascent proteins in LAP1 E482A fibroblasts (estimated in relation to control fibroblasts). Upon cell culture in the presence of puromycin, whole cell lysates were analyzed by immunoblotting using a specific antibody against puromycin to measure the protein synthesis rate; a representative blot is shown. **(c)** Relative levels of insoluble/total protein ratio in LAP1 E482A fibroblasts (estimated in relation to control fibroblasts). Total protein and detergent-insoluble protein extracts subjected to SDS–PAGE were analyzed by BlueSafe staining of polyacrylamide gels to measure protein aggregation levels; a representative gel is shown. **(d)** Relative Nrf2 protein levels at baseline (0 μM) and upon cell exposure to 100 and 200 μM of H_2_O_2_ for 24 h in control and LAP1 E482A fibroblasts (estimated in relation to untreated control fibroblasts). After H_2_O_2_ treatment, whole cell lysates were analyzed by immunoblotting using a specific antibody against Nrf2 to measure the induction of cellular antioxidant defense mediated by Nrf2; a representative blot is shown. Quantitative data are presented as mean ± SEM (*n* = 4 (**b**–**d**) or *n* = 5 (**a**)). Ponceau S staining was used as protein loading control for data normalization before determining relative protein levels (**a**, **b**, **d**). ^§^*p* < 0.05, ^§^^§^*p* < 0.01 and ^§^^§^^§^*p* < 0.001 for comparisons between untreated and treated control fibroblasts using the one-way ANOVA test followed by the Dunnett's multiple comparisons test (**a**) or the Kruskal–Wallis test followed by the Dunn's multiple comparisons test (**d**). ^#^^#^*p* < 0.01, ^#^^#^^#^*p* < 0.001 and ^#^^#^^#^^#^*p* < 0.0001 for comparisons between untreated and treated LAP1 E482A fibroblasts using the one-way ANOVA test followed by the Dunnett's multiple comparisons test (**a**, **d**). *p < 0.05, **p < 0.01, ***p < 0.001 and ****p < 0.0001 for comparisons between control and LAP1 E482A fibroblasts under the same experimental condition using the unpaired *t*-test (**a**–**d**). γ-H2AX, histone variant H2AX phosphorylated at Ser139; ANOVA, Analysis of Variance; DDR, DNA damage response; DNA, deoxyribonucleic acid; H_2_O_2_, hydrogen peroxide; LAP1, lamina-associated polypeptide 1; Nrf2, nuclear factor erythroid 2-related factor 2; SDS–PAGE, sodium dodecyl sulfate–polyacrylamide gel electrophoresis; SEM, standard error of the mean.

To further validate the results of the cluster-specific bioinformatic analysis of LC–MS/MS data described in subsection 3.2, several *in vitro* experiments were performed to verify the deregulation of selected biological processes in LAP1 E482A fibroblasts (Fig. 6); for this purpose, we focused on those clusters linked to physiological functions that are common to most cell types (i.e. DNA repair, mRNA degradation, proteostasis and glutathione metabolism/response to oxidative stress), which could be directly evaluated in the fibroblast cell lines (as opposed to the tissue-specific processes of myogenesis and neurogenesis). Firstly, considering the functional association of various differentially expressed proteins to DNA repair (Fig. 4e; Supplementary Tables S16 and S24), we evaluated the degree of DNA damage in LAP1 E482A fibroblasts at baseline and after treatment with a DNA-damaging agent, consisting in a short exposure to 25 and 50 μg/mL of bleomycin for 30 min, followed by a recovery period of 6 h (Fig. 6a). Whole cell lysates of non-treated and bleomycin-exposed LAP1 E482A and control fibroblasts were analyzed by immunoblotting to determine the levels of histone variant H2AX phosphorylated at Ser139 (γ-H2AX), a DNA damage marker (Fig. 6a). Regarding its DNA damage-inducing effects, the bleomycin treatment caused a significant dose-dependent increase in γ-H2AX levels in both control and LAP1 E482A fibroblasts relatively to respective untreated cells (Fig. 6a). When comparing the two cell lines, we found that the basal levels of DNA damage were significantly augmented (around 34.3 %) in LAP1 E482A *versus* control fibroblasts (Fig. 6a). Upon exposure to similar bleomycin concentrations, LAP1 E482A fibroblasts also exhibited a greater content of DNA damage in relation to control fibroblasts, which is particularly evidenced by the significant increment (approximately 75.1 %) in γ-H2AX levels following treatment with 50 μg/mL of bleomycin (Fig. 6a).

Moreover, because some differentially expressed proteins are involved in the regulation of mRNA degradation (Fig. 4f; Supplementary Table S18), we decided to monitor translation in LAP1 E482A fibroblasts (Fig. 6b), assuming that alterations in mRNA stability can influence protein production. To measure the overall rate of protein synthesis in steady-state conditions, control and LAP1 E482A fibroblasts were incubated with puromycin, after which whole cell lysates were subjected to immunoblotting analysis to assess the levels of puromycylated peptides (Fig. 6b). The results showed that the global protein synthesis rate was significantly elevated (about 94.4 %) in LAP1 E482A fibroblasts comparatively to control fibroblasts (Fig. 6b).

Since the *in silico* approach also unveiled proteostasis-related roles for several deregulated proteins identified in LAP1 E482A fibroblasts (Fig. 4a; Supplementary Table S6), this prompted us to evaluate their intracellular content of insoluble proteins (Fig. 6c). Total protein extracts and detergent-insoluble protein fractions obtained from control and LAP1 E482A fibroblasts grown in standard conditions were analyzed through BlueSafe staining of polyacrylamide gels containing both types of samples (Fig. 6c). We detected a significant increase (around 46.1 %) in the insoluble/total protein ratio, suggestive of protein aggregation, in LAP1 E482A fibroblasts as compared to control fibroblasts (Fig. 6c).

In addition, given the discovery of differentially expressed proteins that modulate the response against oxidative stress (Fig. 4c; Supplementary Table S8), we investigated the induction of the antioxidant defense in LAP1 E482A fibroblasts at baseline and upon incubation with 100 and 200 μM of hydrogen peroxide (H_2_O_2_) for 24 h (Fig. 6d). The protein levels of nuclear factor erythroid 2-related factor 2 (Nrf2), a key transcriptional regulator of the antioxidant response, were assessed in whole cell lysates of untreated and H_2_O_2_-exposed LAP1 E482A and control fibroblasts by immunoblotting analysis (Fig. 6d). The results revealed that the H_2_O_2_ treatment had variable effects depending on the cell line; specifically, the exposure of LAP1 E482A fibroblasts to either H_2_O_2_ concentration used caused a significant elevation in Nrf2 protein levels relatively to non-treated cells, whereas only the highest H_2_O_2_ dose had a significant impact on Nrf2 induction in the case of control fibroblasts (Fig. 6d). When establishing a comparison between the two cell lines, it was possible to observe that, in unstressed conditions, Nrf2 protein levels were already significantly augmented (almost 38.6 %) in LAP1 E482A *versus* control fibroblasts (Fig. 6d). The difference between these cell lines was even more pronounced when they were exposed to 100 μM of H_2_O_2_, with LAP1 E482A fibroblasts exhibiting substantially higher Nrf2 protein levels (approximately 164.1 %) than control fibroblasts (Fig. 6d).

Overall, these experimental findings are consistent with the LC–MS/MS and bioinformatics data, in the sense that they support the existence of alterations in diverse molecular mechanisms in LAP1 E482A fibroblasts, including DDR/DNA repair, mRNA decay/protein synthesis, proteostasis and oxidative stress response, to which the deregulated proteins identified in these cells are functionally associated.

### 3.5 Restoration of LAP1 protein expression in LAP1-deficient human fibroblasts and evaluation of the consequent impact on differentially expressed proteins and deregulated biological processes

**Fig. 7:**
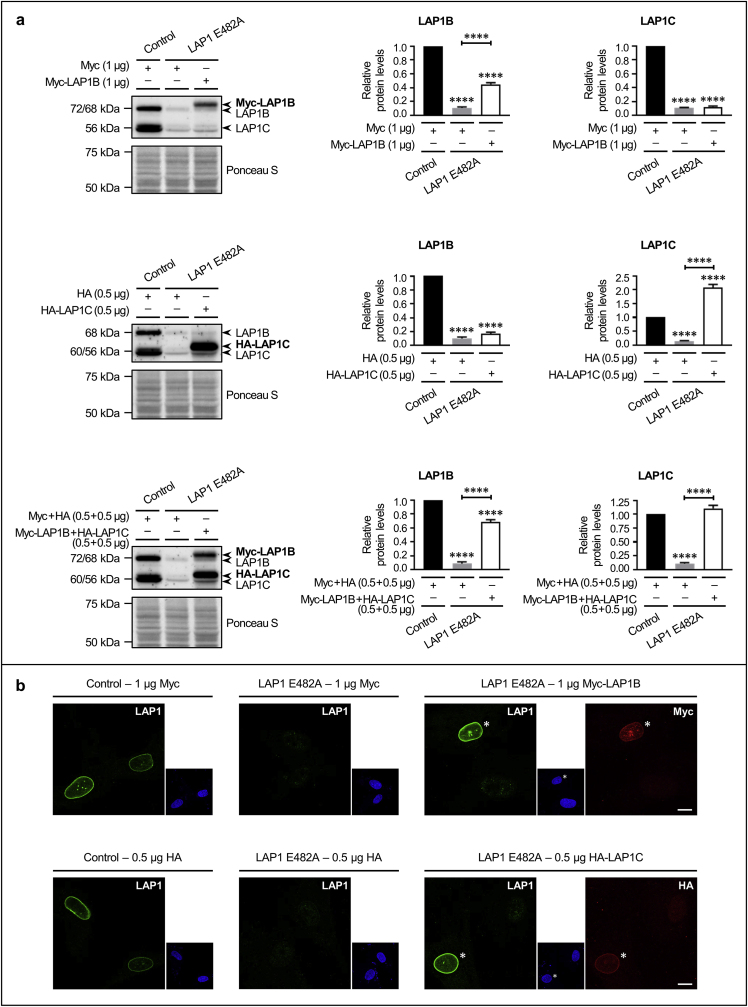
Rescue of LAP1's protein levels and NE localization in LAP1 E482A fibroblasts. **(a)** Relative LAP1B and LAP1C protein levels upon transient transfection of LAP1 E482A fibroblasts with DNA constructs encoding Myc-LAP1B (1 μg; upper panel), HA-LAP1C (0.5 μg; middle panel) or both fusion proteins (0.5 + 0.5 μg; lower panel), or with the corresponding empty expression vectors, during 24 h (estimated in relation to control fibroblasts transfected with pCMV-Myc, pCMV-HA or both expression vectors). Following transfection, whole cell lysates were analyzed by immunoblotting using a specific antibody against LAP1 to assess the recovery of normal LAP1B and LAP1C protein levels; a representative blot is shown for each type of transfection. Quantitative data are presented as mean ± SEM (*n* = 4 (lower panel) or *n* = 5 (upper and middle panels)). Ponceau S staining was used as protein loading control for data normalization before determining relative protein levels. ****p < 0.0001 for comparisons between transfected control and LAP1 E482A fibroblasts using the one-way ANOVA test followed by the Tukey's multiple comparisons test. **(b)** Immunolocalization of LAP1 (green) and transfected Myc-LAP1B (red; upper panel) or HA-LAP1C (red; lower panel) upon transient transfection of LAP1 E482A fibroblasts with the corresponding LAP1B- or LAP1C-encoding DNA construct (1 μg or 0.5 μg, respectively) during 24 h. Immunolocalization of endogenous LAP1 (green) in control and LAP1 E482A fibroblasts transfected with pCMV-Myc (upper panel) or pCMV-HA (lower panel) expression vectors is also shown. Following transfection, cells were fixed, immunostained with LAP1- and/or Myc-/HA-specific primary antibodies linked to AF 488- and/or AF 594-conjugated secondary antibodies, respectively, and incubated with mounting medium containing DAPI (blue). Image acquisition was achieved using an LSM 880 confocal laser scanning microscope with Airyscan; representative microphotographs (one section in the Z-axis) are shown for each type of transfection. Asterisks denote nuclei of transfected LAP1 E482A fibroblasts expressing Myc-LAP1B (upper panel) or HA-LAP1C (lower panel) fusion proteins. Scale bars, 10 μm. AF, Alexa Fluor; ANOVA, Analysis of Variance; DAPI, 4′,6-diamidino-2-phenylindole; DNA, deoxyribonucleic acid; HA, hemagglutinin; LAP1, lamina-associated polypeptide 1; SEM, standard error of the mean. (For interpretation of the references to colour in this figure legend, the reader is referred to the Web version of this article.)

After the functional validation of proteomics results, we carried out transient transfection experiments to reestablish the protein levels and intracellular localization of LAP1B and/or LAP1C isoforms in LAP1 E482A fibroblasts (Fig. 7). This cell line was transfected with different DNA constructs to express either N-terminally Myc-tagged LAP1B, N-terminally hemagglutinin (HA)-tagged LAP1C or both fusion proteins. As control experimental conditions, LAP1 E482A and control fibroblasts were transfected with pCMV-Myc and/or pCMV-HA expression vectors. Upon 24 h of transfection, cells were harvested and the restoration of LAP1 protein levels in LAP1 E482A fibroblasts was verified by immunoblotting using a LAP1-specific antibody to detect endogenous and transfected LAP1 isoforms (Fig. 7a). We were able to partially rescue LAP1B (Fig. 7a, upper panel) and fully rescue LAP1C (Fig. 7a, middle panel) protein expression through single transfections, as well as to almost recover simultaneously the normal protein levels of LAP1B and LAP1C via co-transfection (Fig. 7a, lower panel). In turn, to evaluate the subcellular distribution of transfected fusion proteins, cells were fixed following transfection, incubated with antibodies against LAP1, Myc and/or HA along with fluorophore-conjugated antibodies and analyzed by confocal microscopy (Fig. 7b). Our results confirmed that, like the endogenous LAP1 protein in control fibroblasts, both Myc-LAP1B (Fig. 7b, upper panel) and HA-LAP1C (Fig. 7b, lower panel) fusion proteins presented a NE localization in transfected LAP1 E482A fibroblasts. Of note, in these rescue experiments, approximately 10 % and 19 % of LAP1 E482A fibroblasts transfected with Myc-LAP1B or HA-LAP1C plasmids, respectively, exhibited a LAP1 immunofluorescent signal that was at least as intense as the LAP1 immunostaining observed in control fibroblasts.

**Fig. 8:**
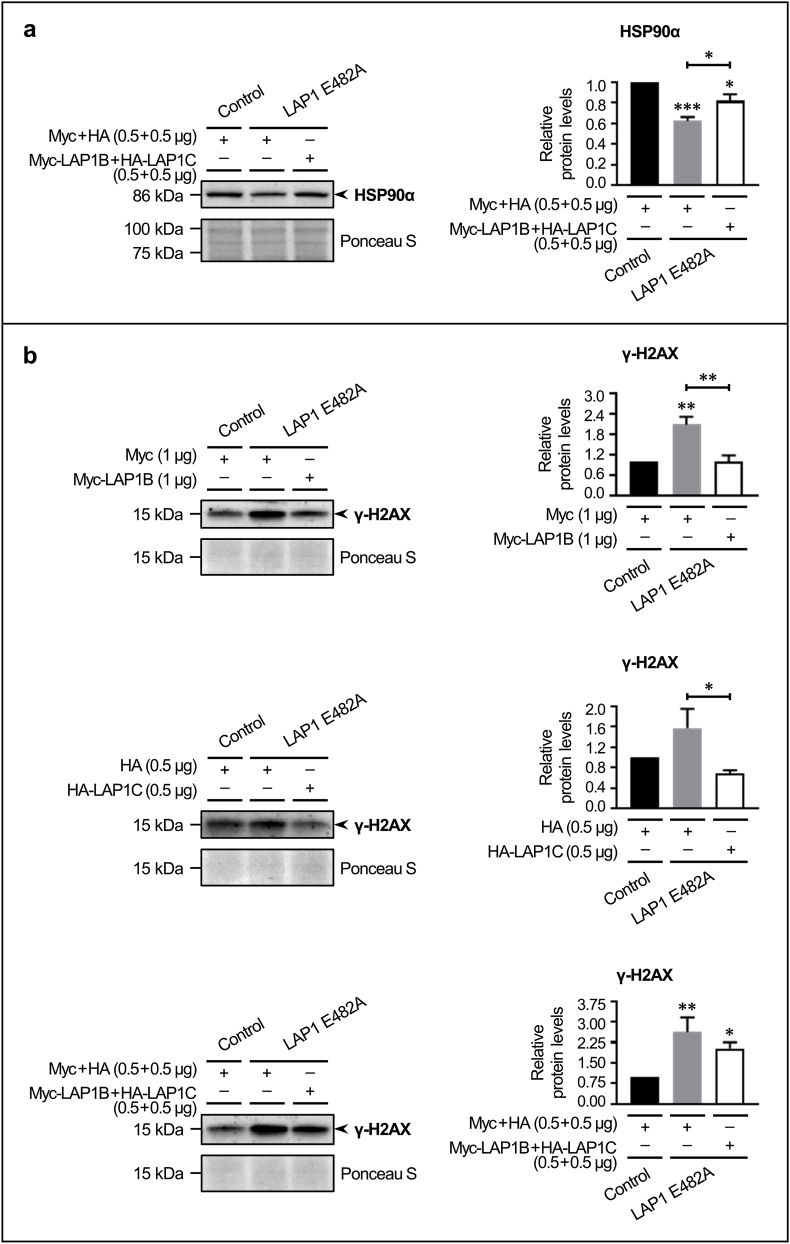
Analysis of the effects of LAP1 rescue on **(a)** the differentially expressed protein HSP90α and **(b)** the deregulated biological process of DDR/DNA repair in LAP1 E482A fibroblasts. **(a)** Relative HSP90α protein levels upon transient transfection of LAP1 E482A fibroblasts with both DNA constructs encoding Myc-LAP1B and HA-LAP1C fusion proteins (0.5 + 0.5 μg), or with the corresponding empty expression vectors, during 24 h (estimated in relation to control fibroblasts transfected with pCMV-Myc and pCMV-HA expression vectors). Following transfection, whole cell lysates were analyzed by immunoblotting using a specific antibody against HSP90α to assess the recovery of its normal protein levels; a representative blot is shown. **(b)** Relative γ-H2AX levels upon transient transfection of LAP1 E482A fibroblasts with DNA constructs encoding Myc-LAP1B (1 μg; upper panel), HA-LAP1C (0.5 μg; middle panel) or both fusion proteins (0.5 + 0.5 μg; lower panel), or with the corresponding empty expression vectors, during 24 h (estimated in relation to control fibroblasts transfected with pCMV-Myc, pCMV-HA or both expression vectors). Following transfection, whole cell lysates were analyzed by immunoblotting using a specific antibody against γ-H2AX to assess the attenuation of DNA damage levels; a representative blot is shown for each type of transfection. Quantitative data are presented as mean ± SEM (*n* = 4 (**a**, **b**–middle panel) or *n* = 5 (**b**–upper and lower panels)). Ponceau S staining was used as protein loading control for data normalization before determining relative protein levels. *p < 0.05, **p < 0.01 and ***p < 0.001 for comparisons between transfected control and LAP1 E482A fibroblasts using the one-way ANOVA test followed by the Tukey's multiple comparisons test (**a**, **b**–upper panel) or the Kruskal–Wallis test followed by the Dunn's multiple comparisons test (**b**–middle and lower panels). γ-H2AX, histone variant H2AX phosphorylated at Ser139; ANOVA, Analysis of Variance; DDR, DNA damage response; DNA, deoxyribonucleic acid; HA, hemagglutinin; HSP90α, heat shock protein 90α family class A member 1; LAP1, lamina-associated polypeptide 1; SEM, standard error of the mean.

Lastly, we investigated if the restoration of LAP1B and/or LAP1C protein expression in LAP1 E482A fibroblasts could revert the abnormal levels of differentially expressed proteins, such as those validated in subsection 3.3 (i.e. HSP90α, caldesmon 1, peroxiredoxin 6 and Fyn), and/or the alterations in deregulated biological processes, particularly in DDR/DNA repair (Fig. 8). We found that, by co-expressing Myc-LAP1B and HA-LAP1C, it was possible to partly attenuate HSP90α downregulation in LAP1 E482A fibroblasts (Fig. 8a). Furthermore, the rescue of either LAP1B (Fig. 8b, upper panel) or LAP1C (Fig. 8b, middle panel) caused a significant reduction in steady-state γ-H2AX levels in LAP1 E482A fibroblasts. A similar tendency towards a decrease in basal γ-H2AX levels was observed when these cells were transfected with both LAP1B- and LAP1C-encoding DNA constructs (Fig. 8b, lower panel). Therefore, these results suggest a causal link between LAP1 deficiency and the described changes in HSP90α protein expression and DNA damage levels.

## 4 Discussion

Mutations in the human *TOR1AIP1* gene, though rare, pose a major threat to cell homeostasis, as reflected by the broad clinical spectrum linked to these genetic alterations that includes limb–girdle muscular dystrophy, dystonia, dilated cardiomyopathy, progeroid-like multisystemic disorder and, more recently, myasthenic syndrome [13,[16], [17], [18], [19], [20], [21], [22], [23], [24]]. Besides its medical relevance due to the pathogenic effects of *TOR1AIP1* mutations, the study of LAP1 is also important from a biological point of view because increasing evidence suggests that this protein is able to participate in many cellular activities, either more universal (e.g. DDR and mitosis) [[5], [6], [7]] or specific for certain cell/tissue types (e.g. chromatin reorganization in spermatids and neuromuscular transmission in skeletal muscle) [[8], [9], [10], [11], [12], [13], [14], [15]]. Given that a complete functional characterization of LAP1 has not yet been achieved, this motivated us to investigate the molecular repercussions of its deficiency in human cells with the ultimate goal of discovering novel putative physiological roles of LAP1 and, hopefully, potential therapeutic targets for *TOR1AIP1*-related pathologies.

Herein, we carried out a quantitative LC–MS/MS analysis of patient-derived LAP1 E482A fibroblasts' proteome. To our knowledge, this is the first work to use a proteomics-based approach to analyze patients’ cells with *TOR1AIP1* mutations in order to assess the overall impact of LAP1 knockdown. This methodology permitted to identify 386 proteins exhibiting a differential expression pattern between LAP1 E482A and control fibroblasts (Fig. 2; Supplementary Tables S1 and S2). Using bioinformatics tools (i.e. PANTHER [40] and STRING [41] online resources), a global analysis of all deregulated proteins found in LAP1 E482A fibroblasts was initially performed (Fig. 3; Supplementary Tables S3 and S4), which proved to be insufficient to elucidate the pathological consequences of LAP1 depletion owing to the great functional diversity of these proteins. So, targeted *in silico* analyses of clusters of interacting proteins were then conducted (Supplementary Tables S6–S35), allowing for a more thorough characterization of the biological processes/signaling cascades potentially altered in LAP1 E482A fibroblasts. Special focus was given in this study to alterations in DNA repair, mRNA degradation/translation, proteostasis and glutathione metabolism/response to oxidative stress, which will be discussed in more detail in the next subsections. Of note, it is worth referring that several differentially expressed proteins detected in these cells are functionally associated to myogenesis/muscle contraction (Fig. 4b; Supplementary Table S7) and neurogenesis (Fig. 4d; Supplementary Table S12), indicating that LAP1 deficiency may additionally interfere with these tissue-specific developmental processes, which agrees well with the fact that the LAP1 E482A mutation is linked to severe cardio/neuropathogenic effects [17].

### 4.1 DNA repair

Our proteomics results revealed a deregulation of DNA repair proteins in LAP1 E482A fibroblasts (Fig. 4e; Supplementary Tables S16 and S24), suggesting that an impaired ability to accurately correct DNA damage may be a pathological mechanism subjacent to the disorders caused by *TOR1AIP1* mutations. Indeed, this hypothesis is supported by experimental data. First, in a previous report describing another *TOR1AIP1* mutation, a mild increase in the number of nuclear foci containing γ-H2AX or p53-binding protein 1 (53BP1) DDR markers was observed in patient-derived fibroblast cell lines relatively to control ones, without prior induction of DNA damage through exogenous sources [23]. Second, in the present study, we also found significantly elevated γ-H2AX levels in LAP1 E482A *versus* control fibroblasts, not only in steady-state conditions but after bleomycin exposure (50 μg/mL) as well (Fig. 6a). In addition, we confirmed that this increased susceptibility to accumulate DNA damage at baseline could be reverted by rescuing LAP1B and/or LAP1C protein expression in LAP1 E482A fibroblasts (Fig. 8b), which shows that LAP1 is required to preserve genome integrity, perhaps by modulating the efficient repair of DNA lesions. Third, and in agreement with this last idea, our group recently uncovered that LAP1 participates in the DDR together with telomeric repeat-binding factor 2 (TRF2) [7], a telomere-resident protein that has been implicated in HRR [43,44] and NHEJ [45] pathways of DNA double-strand break (DSB) repair at extra-telomeric chromatin sites. In short, we discovered that LAP1:TRF2 interaction in human cells is induced after exposure to DNA-damaging agents and is regulated by protein phosphorylation. Under such genotoxic conditions, their nuclear protein levels rise and they co-localize in the nucleus with γ-H2AX-positive DSB foci as well as with ataxia–telangiectasia mutated (ATM) protein [7], a pivotal DDR kinase that phosphorylates LAP1 [46] and TRF2 [46,47] upon DNA damage. Future investigation will be needed to characterize the putative role of LAP1 in DDR and decipher if its dysfunction may disrupt a particular or various DNA repair signaling cascades. Still, some hypotheses regarding possible repercussions of LAP1 deficiency on the functioning of DNA repair mechanisms can be proposed based on the results of our bioinformatic analysis, as follows.

BER [48] and DNA single-strand break (SSB) repair (SSBR) [49] are two overlapping pathways that repair oxidized bases and reactive oxygen species (ROS)-induced SSBs, respectively, among other types of DNA damage. X-ray repair cross-complementing protein 1 (XRCC1) is a key coordinator of nuclear BER/SSBR in virtue of its ability to bind to multiple DNA repair proteins that act in every step of these signaling cascades [50]. The finding that XRCC1 is upregulated in LAP1 E482A fibroblasts (Fig. 2a; Supplementary Table S1) could be indicative of BER/SSBR hyperactivation as a compensatory mechanism to counteract the negative effects of intracellular ROS accumulation on DNA structure. As it will be discussed in subsection 4.4, alterations in proteins involved in glutathione metabolism and oxidative stress response were detected in LAP1 E482A fibroblasts (Fig. 4, Fig. 6d; Supplementary Table S8) and we postulate that there could be an intimate association between a putative redox imbalance and the occurrence of oxidative DNA damage in these cells. Interestingly, XRCC1 interacts with and stimulates 8-oxoguanine DNA glycosylase 1 (OGG1), a BER enzyme that specifically recognizes and triggers the repair of 7,8-dihydro-8-oxoguanine (8-oxoG), a ROS-induced mutagenic DNA base lesion [51]. Moreover, it has been reported that DNA polymerase β (Pol β), an important gap-filling DNA polymerase in BER [48] and SSBR [49], is recruited by XRCC1 to oxidative SSBs [52]. Another remarkable observation is that human *XRCC1* mutations have been linked to cerebellar ataxia and axonal neuropathy, and the resulting marked reduction of XRCC1 protein is coupled to defective SSBR [53]. Hence, XRCC1's action in this DNA repair pathway may be neuroprotective, which is in accordance with the fact that the brain is highly susceptible to oxidative stress [54]. Overall, these data support the idea that XRCC1 upregulation in LAP1 E482A fibroblasts may reflect an increased need to repair oxidative DNA lesions and raise the possibility that, if the mechanisms that normally correct such damage eventually fail, this could contribute to the neurological dysfunction seen in some patients affected by *TOR1AIP1* mutations [17,23,24].

A second hypothetical scenario that can be envisaged is NHEJ impairment in LAP1 E482A fibroblasts. Besides cooperating with several proteins in the resolution of DSBs via NHEJ-mediated repair [55], X-ray repair cross-complementing protein 4 (XRCC4) is mostly recognized by its ability to bind, stabilize and induce the activity of DNA ligase IV (Lig4) [56,57], the only eukaryotic DNA ligase that has been found to operate in NHEJ [58]. In agreement with XRCC4 being a core component of this DNA repair pathway, human *XRCC4* mutations leading to greatly reduced protein levels of XRCC4 (and, consequently, Lig4) have been correlated with hypersensitivity to DNA-damaging agents due to NHEJ defects [59,60]. As such, XRCC4 downregulation in LAP1 E482A fibroblasts (Fig. 2b; Supplementary Table S2) is predicted to have a prominent negative effect on the efficiency of the main DSB repair mechanism. Noteworthy, *XRCC4* mutations have been identified in individuals displaying global growth failure and microcephaly, apart from other variable clinical symptoms [59,60]. This phenotype resembles certain features of the multisystemic syndrome presented by some patients with *TOR1AIP1* mutations, who also suffered from severe/moderate microcephaly and growth retardation [23,24], suggesting that faulty functioning of NHEJ may be a contributing factor for this devastating pathology.

### 4.2 mRNA degradation and translation

Based on our proteome analysis, the deregulation of deadenylation-dependent mRNA decay (Fig. 4f; Supplementary Table S18) emerged as another putative pathological process linked to LAP1 deficiency. For most eukaryotic mRNAs, deadenylation-dependent decay is the primary mechanism of default turnover of stable transcripts, wherein shortening of the 3′ poly(A) tail (i.e. deadenylation) of mRNAs serves as a major trigger for their degradation [61]. Several proteins that coordinate this key first step in mRNA turnover, namely carbon catabolite repression 4–negative on TATA-less (CCR4–NOT) transcription complex subunits 4 and 8 (CNOT4 and CNOT8) as well as poly(A)-specific ribonuclease (PARN), are downregulated in LAP1 E482A fibroblasts (Fig. 2b; Supplementary Table S2). Both PARN [62,63] and CNOT8, the latter of which acts within the CCR4–NOT complex [64,65], have intrinsic deadenylase activity and catalyze 3′ poly(A) tail cleavage of cytoplasmic transcripts. CNOT4, in turn, is an E3 ubiquitin–protein ligase that interacts with the CCR4–NOT complex [64,66], possibly functioning as a regulatory factor that enhances its ability to elicit mRNA decay [66]. So, one can postulate that there might be a decline in mRNA deadenylation events in LAP1 E482A fibroblasts, leading to suppression of the main mRNA degradation mechanism; this may culminate in the stabilization of some translatable mRNAs and, ultimately, in higher abundance levels of the proteins produced from such transcripts.

Importantly, we verified experimentally that LAP1 E482A fibroblasts exhibit an abnormally increased rate of global protein synthesis as compared to control fibroblasts (Fig. 6b), which appears to be consistent with the above hypothesis. In fact, it is widely acknowledged that there is an intimate association between mRNA turnover and translation [61]. This can be exemplified by the actions of PARN; besides stimulating mRNA deadenylation [62,63], PARN may function in translation repression by competing with eukaryotic initiation factor 4E (eIF4E) for binding to the 5′ cap structure of mRNAs, preventing the initiation of cap-dependent translation [67]. CNOT4 plays a role as a negative translational regulator as well, being involved in the inhibition of global translation during nutrient starvation and that of transcripts causing ribosome stalling. The relevance of CNOT4 as a translational repressor is highlighted by the observation that its depletion impedes an efficient attenuation of protein synthesis under stress conditions, which is accompanied by protein aggregation [68]. Thus, it seems possible that the downregulation of PARN and CNOT4 in LAP1 E482A fibroblasts may contribute to the described alterations in translation. Of note, CNOT4 is also a critical modulator of proteolytic degradation by the ubiquitin–proteasome system (UPS), being required for the correct assembly and activity of the 26S proteasome [69,70], and the clearance of aberrant proteins [70,71]. Another significant function of this E3 ubiquitin–protein ligase in co-translational protein quality control (PQC) refers to its ability to ubiquitinate nascent polypeptide-associated complex (NAC) [72,73], a ribosome-bound chaperone that interacts with emerging proteins to assist their initial folding and avoid premature aggregation during translation [74]. It has been proposed that CNOT4-mediated ubiquitination of NAC promotes its association with both the ribosome and the proteasome, which may serve to target abnormally folded or other defective nascent proteins for degradation [73]. Taken together, these data further suggest that CNOT4 downregulation in LAP1 E482A fibroblasts may affect proteostasis, another process potentially impaired in these cells, as discussed below.

### 4.3 Proteostasis

In 2014, Dorboz et al. reported that LAP1 E482A fibroblasts contain intracellular aggregates of mutant LAP1 [17], which could be a sign of a pre-existing imbalance in PQC mechanisms involved in the disposal of defective proteins. The present study provides additional experimental evidence of the loss of protein homeostasis in LAP1 E482A fibroblasts, as we found a significantly augmented insoluble/total protein ratio in these cells relatively to control fibroblasts, compatible with a protein aggregation phenotype (Fig. 6c). In line with this, our proteomics results revealed that various key PQC factors acting at different stages to safeguard the proteome (i.e. foldase/holdase chaperones and UPS components) are deregulated in LAP1 E482A fibroblasts; among these are α-crystallin B chain (αB-crystallin), HSP90α, HSP family A member 2 (HSPA2), HSP family B member 6 (HSPB6), stress-induced phosphoprotein 1 (STIP1) homology and U-box-containing protein 1 (STUB1), and ubiquitin-activating enzyme E1 (UBA1) (Fig. 4a; Supplementary Table S6).

αB-crystallin and HSPB6 are members of the small HSP (sHSP) family of chaperones, the first line of defense against irreversible protein aggregation [75]. During proteotoxic stress, αB-crystallin [76,77] and HSPB6 [78] prevent unfolding proteins from aggregating by holding them in a folding-competent soluble state that enables spontaneous or chaperone-assisted renaturation. Moreover, they form soluble complexes with intrinsically disordered and amyloid-forming proteins to inhibit oligomerization/fibrillation and precipitation into insoluble aggregates, abrogating their cytotoxicity [79,80]. Our finding that αB-crystallin and HSPB6 are upregulated in LAP1 E482A fibroblasts (Fig. 2a; Supplementary Table S1) hints at an increased susceptibility of cellular proteins to undergo misfolding and aggregate, possibly due to endogenous insults (e.g. oxidative stress). Curiously, αB-crystallin exhibits high binding affinity for the redox-active copper (Cu^2+^) ion, a cofactor of several enzymes, and is able to suppress Cu^2+^-induced ROS generation and protein aggregation [81,82]. It has also been shown that αB-crystallin [83] and HSPB6 [84] are upregulated in response to oxidative stress. Hence, it is conceivable that their upregulation in LAP1 E482A fibroblasts could be related with their redox attenuation and stress resistance properties, reinforcing the idea that these cells may be more predisposed to accumulate ROS, a well-recognized damage source that disrupts the native structure of and inactivates proteins [85].

In turn, HSPA2 and HSP90α belong, respectively, to the HSP70 and HSP90 chaperone families that regulate protein (re)folding, disaggregation and degradation [86,87]. HSP90 is known to promote *de novo* protein folding and the stabilization of signaling clients in a semi-folded conformation required for ligand binding [88,89]. In addition, HSP90 [90,91] and HSPA2 [92] possess anti-aggregation and pro-refolding properties under denaturing conditions. This leads us to hypothesize that the downregulation of HSP90α and HSPA2 in LAP1 E482A fibroblasts (Fig. 2b; Supplementary Table S2) may affect the productive folding of nascent proteins and chaperone-mediated refolding of stress-denatured ones, intensifying the cellular demand for the potent buffering activity of αB-crystallin and HSPB6. Furthermore, HSP90 can target (non-)functional mature clients and unfolded proteins towards destruction by the UPS via cooperation with STUB1 [93,94], a master regulator of protein turnover. The latter has a dual role as an E3 ubiquitin–protein ligase [94] and as a co-chaperone for HSP70 [95] and HSP90 [93] family members, establishing a link between these HSPs and the 26S proteasome. STUB1 negatively modulates their chaperone activity [93,95] and ubiquitinates HSP70-/HSP90-bound substrates, switching their fate from chaperone-assisted (re)folding to proteasomal degradation [93,94]. UBA1 is also an important player in the removal of abnormal proteins by the UPS [96], whose function involves the activation of ubiquitin to be posteriorly attached to proteins targeted for proteolysis [97,98]. Considering these data, another deleterious consequence of the downregulation of HSP90α, STUB1 and UBA1 in LAP1 E482A fibroblasts (Fig. 2b; Supplementary Table S2) likely includes the impairment of UPS-regulated elimination of misfolded or damaged proteins, which may create an aggregation-prone intracellular environment, as supported by our experimental results.

Remarkably, HSP90α protein levels were partially recovered after rescuing the normal expression of both LAP1 isoforms in LAP1 E482A fibroblasts (Fig. 8a), revealing an association between LAP1 deficiency and HSP90α downregulation that could indicate a yet-unknown role for LAP1 in proteostasis. It is well established that loss of proteome integrity accelerates aging and the development of neurodegenerative and cardiac diseases, among others [99]. In accordance with this, the LAP1 E482A mutation causes a severe phenotype combining heart (cardiomyopathy) and brain (dystonia and cerebellar atrophy) dysfunction [17]. Other *TOR1AIP1* mutations manifest as a progeroid-like multisystemic condition (including, but not limited to, progressive neurological deterioration, cardiac/skeletal muscle defects and cataracts) [23,24] or as striated muscle disorders [13,16,[18], [19], [20], [21], [22]]. Similarly, the deregulation of the above PQC factors has been linked, for instance, to premature senescence (STUB1) [100], neurodegenerative motor neuron disease (UBA1) [101], myofibrillar myopathy, cardiomyopathy and congenital cataracts (αB-crystallin) [102]. It is interesting to note the similarities between LAP1 and these proteins regarding their associated pathologies, which affect tissues composed of long-lived, non-proliferating cells that are especially reliant on a permanent, fine-tuned regulation of the cellular proteome to preserve their functionality. Altogether, the presented findings allow the speculation that a reduction in proteostasis efficiency may have an important contribution, perhaps even more significantly in tissues containing post-mitotic cells, to the pathological phenotypes arising from mutations that largely abolish LAP1 expression, as the one studied here.

### 4.4 Glutathione metabolism and response to oxidative stress

In this work, the experimental and bioinformatics results revealed alterations in the redox status and functioning of antioxidant defense systems in LAP1 E482A fibroblasts. We detected significantly elevated Nrf2 protein levels in LAP1 E482A *versus* control fibroblasts without prior exposure to stressful agents (Fig. 6d). A higher sensitivity to treatment with exogenous H_2_O_2_ (100 μM) was also observed in LAP1 E482A fibroblasts, as shown by a substantial Nrf2 accumulation in these cells that contrasts with the slight increase in Nrf2 protein levels in control fibroblasts (Fig. 6d). It is known that constitutively expressed Nrf2 is constantly degraded and maintains a low basal protein level in a physiological context; however, in response to oxidative/electrophilic stress, *de novo* synthesized Nrf2 is stabilized to activate the transcription of genes encoding cytoprotective antioxidant and detoxifying enzymes, such as nicotinamide adenine dinucleotide (phosphate) (NAD(P)H) quinone oxidoreductase 1 (NQO1) [103]. This ROS-inducible enzyme is involved in the cellular defense against oxidative stress, for example by reducing ubiquinone, a constituent of the mitochondrial electron transport chain, to its antioxidant form [104] as well as scavenging ROS [105]. Interestingly, NQO1 is one of the upregulated proteins identified by LC–MS/MS in LAP1 E482A fibroblasts (Fig. 2a; Supplementary Table S1), which is consistent with Nrf2 induction in a basal state (Fig. 6d). These data could indicate that the amount of ROS normally present in these cells may exceed a physiological level and cause oxidative stress, leading to hyperactivation of endogenous antioxidant mechanisms, like the Nrf2-mediated stress response, to shift the redox equilibrium back to a more reducing state. Our proteomics analysis also evidenced a deregulation of proteins implicated in glutathione metabolism, namely γ-glutamyl transferase 5 (GGT5) and glutathione synthetase (GS), in LAP1 E482A fibroblasts (Fig. 4c; Supplementary Table S8). Glutathione is a potent antioxidant agent abundant in the cytosol, where it exists mostly in a reduced form (i.e. GSH) under homeostatic redox conditions; when this balance is perturbed by oxidants, glutathione is temporarily converted to an oxidized form (e.g. GSSG) while carrying out antioxidant functions that include, for instance, protecting thiol groups in proteins from permanent oxidation, neutralizing ROS and detoxifying xenobiotics [106]. The fact that GS, the enzyme that catalyzes the second and final step in glutathione synthesis [107], is upregulated in LAP1 E482A fibroblasts (Fig. 2a; Supplementary Table S1) points to a stimulation of glutathione production. In addition to *de novo* biosynthesis, another means to maintain an elevated intracellular concentration of glutathione is by enhancing the uptake of exogenous one, which can be promoted by GGT5 [108]. Its hydrolytic action initiates the extracellular degradation of glutathione S-conjugates, GSH and GSSG [109], releasing intermediates that are further metabolized so that precursor amino acids can be taken up by cells and reincorporated into glutathione [108]. So, the upregulation of GS and GGT5 in LAP1 E482A fibroblasts (Fig. 2a; Supplementary Table S1) may reflect an adaptive response to amplify the cells’ capacity to replenish the glutathione pool in the intracellular milieu and maximize the redox potential when facing oxidative challenges, namely by supporting a higher activity of glutathione-dependent protective enzymes, as is the case of glutathione S-transferase μ4 (GSTM4), another protein upregulated in these cells (Fig. 2a; Supplementary Table S1).

Among the factors that possibly contribute to the increased predisposition of LAP1 E482A fibroblasts to develop oxidative stress at baseline might be the deregulation of several metabolite interconversion enzymes, including cytochrome P450 family 1 subfamily B member 1 (CYP1B1), microsomal glutathione S-transferase 1 (MGST1) and peroxiredoxin 6 (Fig. 4c; Supplementary Table S8). CYP1B1 catalyzes the oxidation of 17β-estradiol (E_2_), converting it mainly into 2-hydroxyestradiol (2-OHE_2_) and 4-hydroxyestradiol (4-OHE_2_) metabolites [110] that can stimulate ROS production and consequent oxidative DNA damage [111,112]. The upregulation of CYP1B1 in LAP1 E482A fibroblasts (Fig. 2a; Supplementary Table S1) may, therefore, culminate in the increased formation of harmful reactive products. On top of that, the concurrent downregulation of peroxiredoxin 6 and MGST1 in these cells (Fig. 2b; Supplementary Table S2) may further exacerbate the redox imbalance by decreasing their capability to detoxify diverse strongly oxidizing substrates. Indeed, peroxiredoxin 6 [113] and MGST1 [114,115] contribute to the maintenance of redox homeostasis by promoting the reduction of H_2_O_2_ and/or organic hydroperoxides. Notably, peroxiredoxin 6 exhibits a unique ability to both reduce phospholipid hydroperoxides [113,116,117] and induce their hydrolysis/reacylation, which permits the complete repair of cell membranes damaged by oxidative stress-mediated lipid peroxidation [116,117]. Moreover, MGST1 confers additional protection against oxidative stress by conjugating GSH to cytotoxic lipid peroxidation products (e.g. 4-hydroxy-2-nonenal (4-HNE)) [115] and xenobiotics [114], yielding metabolites with attenuated reactivity. Regarding possible repercussions of peroxiredoxin 6 downregulation, it has been found that *Prdx6*^*−/−*^ cells/tissues present an elevated content of ROS and protein oxidation products even in unstressed conditions [118], as well as abnormally high lipid peroxidation levels when subjected to oxidative stress due to defective repair of oxidized cell membranes [116,117]. In the case of MGST1, its depletion can also lead to increased lipoperoxidation in cell membranes [119]. Globally, these observations support our hypothesis that a disturbance in redox homeostasis may be intimately associated with the higher susceptibility to DNA damage (Fig. 6a) and propensity to protein aggregation (Fig. 6c) that characterize LAP1 E482A fibroblasts and additionally indicate that lipid peroxidation may be another pathogenic event occurring in these cells. By damaging such important biomolecules, oxidative stress has a major role in the genesis of cell dysfunction and may facilitate the progression of *TOR1AIP1*-related disorders, as reported for a multitude of other human diseases [120]. Importantly, considering that neurons [54], cardiomyocytes [121] and skeletal myofibers [122] have exceedingly high metabolic rates to maintain the intense level of activity of the brain, heart and skeletal muscle—a feature turning these cell types into major factories of ROS—, the inability to efficiently counteract a pro-oxidative intracellular environment, as appears to happen in LAP1 E482A fibroblasts, may disrupt their normal functioning and potentially contribute to the high frequency of pathological phenotypes affecting these tissues in patients with *TOR1AIP1* mutations.

## 5 Conclusions

Since its discovery in 1988 [1], the precise function of LAP1 has remained elusive. Thenceforth, several and quite distinct biological roles have been proposed [[5], [6], [7], [8], [9], [10], [11], [12], [13], [14], [15]] and the present study further highlights the functional complexity of LAP1. The quantitative proteome characterization of patient-derived LAP1-depleted fibroblasts and its comparison to control cells permitted the identification of numerous differentially expressed proteins, belonging to the most diverse protein classes, and related signaling pathways potentially deregulated in response to LAP1 dysfunction. Besides giving additional support to the described involvement of LAP1 in DDR/DNA repair [7], our work unveils a possible link between LAP1 deficiency and the deregulation of other vital cellular processes, such as mRNA decay/translation, proteostasis and glutathione metabolism/response to oxidative stress. Through an integrated analysis of the known functions of various differentially expressed proteins, putative mechanisms underlying certain pathogenic features observed in patients lacking a normal LAP1 expression were suggested, providing new clues about LAP1's functional properties that can inspire future mechanistic studies. In addition, we expect that the findings reported here regarding the biological processes/signaling pathways altered in LAP1 E482A fibroblasts can be validated by additional research groups that investigate other *TOR1AIP1* mutations, which would contribute to the elucidation of the pathological mechanisms subjacent to these rare diseases. Importantly, our proteomics dataset may also be a useful resource for the discovery of potential therapeutic strategies to ameliorate the severity and/or delay the progression of *TOR1AIP1*-associated nuclear envelopathies. Of these, the pharmacological induction of the endogenous antioxidant defense system [120] might well prove to be a valuable approach, given the negative impact of oxidative stress on genome and proteome integrity [123], allied to the fact that both DNA repair and proteostasis networks seem to be compromised in LAP1-deficient cells, according to our results. In summary, this work may significantly advance our understanding of LAP1's pathophysiological significance.

## Consent for publication

Not applicable.

## Ethical approval and consent for participation

Not applicable.

## Data availability

The data that support the findings of this study are included in the published article and its Supplementary Data file. The MS proteomics data generated at the CRG/UPF Proteomics Unit have been deposited to the ProteomeXchange consortium [38] via the PRIDE [39] partner repository, having the dataset identifier PXD035200.

## CRediT authorship contribution statement

**Cátia D. Pereira:** Writing – review & editing, Writing – original draft, Methodology, Investigation, Funding acquisition, Formal analysis, Conceptualization, Visualization. **Guadalupe Espadas:** Writing – review & editing, Writing – original draft, Investigation, Formal analysis. **Filipa Martins:** Writing – review & editing, Methodology, Funding acquisition, Conceptualization. **Anne T. Bertrand:** Writing – review & editing, Resources. **Laurent Servais:** Writing – review & editing, Resources. **Eduard Sabidó:** Writing – review & editing, Resources. **Philippe Chevalier:** Writing – review & editing. **Odete A.B. da Cruz e Silva:** Writing – review & editing, Funding acquisition, Resources. **Sandra Rebelo:** Writing – review & editing, Supervision, Resources, Methodology, Funding acquisition, Conceptualization.

## Declaration of competing interest

The authors declare that they have no known competing financial interests or personal relationships that could have appeared to influence the work reported in this paper.

## Figures and Tables

**figs1:**
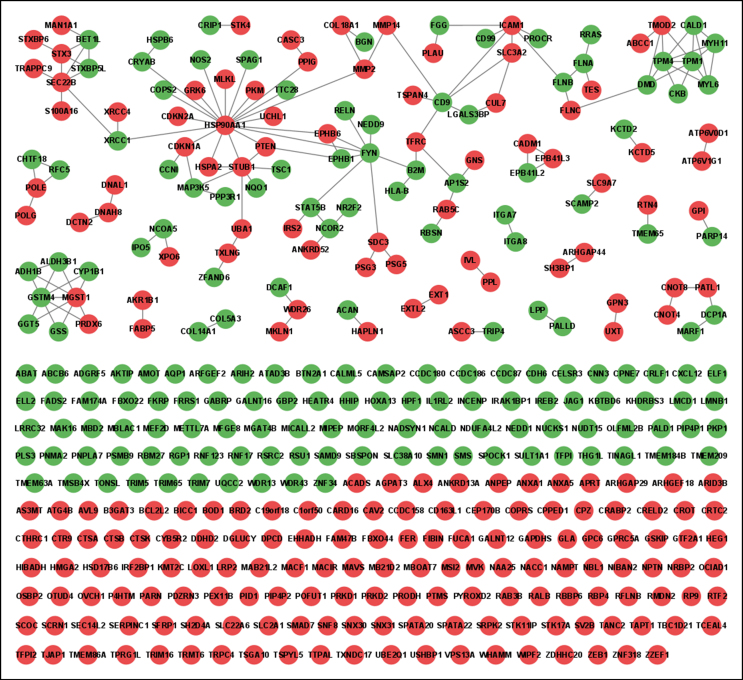


**figs2:**